# Social contact patterns during the early COVID-19 pandemic in Norway: insights from a panel study, April to September 2020

**DOI:** 10.1186/s12889-024-18853-8

**Published:** 2024-05-29

**Authors:** Lamprini Veneti, Bjarne Robberstad, Anneke Steens, Frode Forland, Brita A. Winje, Didrik F. Vestrheim, Christopher I. Jarvis, Amy Gimma, W. John Edmunds, Kevin Van Zandvoort, Birgitte Freiesleben de Blasio

**Affiliations:** 1 Department of Infection Control and Preparedness, Norwegian Institute of Public Health, Lovisenberggata 8, Oslo, 0456 Norway; 2 Department of Global Public Health and Primary Care, University of Bergen, Bergen, Norway; 3 Department of Infection Control and Vaccine, Norwegian Institute of Public Health, Oslo, Norway; 4 Centre for Mathematical Modelling of Infectious Diseases, Department of Infectious Disease Epidemiology, London School of Hygiene & Tropical Medicine, London, UK; 5 Department of Method Development and Analytics, Norwegian Institute of Public Health, Oslo, Norway; 6 Oslo Center for Biostatistics and Epidemiology, Department of Biostatistics, Institute of Basic Medical Sciences, University of Oslo, Oslo, Norway

**Keywords:** COVID-19, Physical distancing, Social distance, Contact pattern, Contact matrices, Control measures, CoMix

## Abstract

**Background:**

During the COVID-19 pandemic, many countries adopted social distance measures and lockdowns of varying strictness. Social contact patterns are essential in driving the spread of respiratory infections, and country-specific measurements are needed. This study aimed to gain insights into changes in social contacts and behaviour during the early pandemic phase in Norway.

**Methods:**

We conducted an online panel study among a nationally representative sample of Norwegian adults by age and gender. The panel study included six data collections waves between April and September 2020, and 2017 survey data from a random sample of the Norwegian population (including children < 18 years old) were used as baseline. The market research company Ipsos was responsible for carrying out the 2020 surveys. We calculated mean daily contacts, and estimated age-stratified contact matrices during the study period employing imputation of child-to-child contacts. We used the next-generation method to assess the relative reduction of R0 and compared the results to reproduction numbers estimated for Norway during the 2020 study period.

**Results:**

Over the six waves in 2020, 5 938 observations/responses were registered from 1 718 individuals who reported data on 22 074 contacts. The mean daily number of contacts among adults varied between 3.2 (95%CI 3.0-3.4) to 3.9 (95%CI 3.6–4.2) across the data collection waves, representing a 67–73% decline compared to pre-pandemic levels (baseline). Fewer contacts in the community setting largely drove the reduction; the drop was most prominent among younger adults. Despite gradual easing of social distance measures during the survey period, the estimated population contact matrices remained relatively stable and displayed more inter-age group mixing than at baseline. Contacts within households and the community outside schools and workplaces contributed most to social encounters. Using the next-generation method R0 was found to be roughly 25% of pre-pandemic levels during the study period, suggesting controlled transmission.

**Conclusion:**

Social contacts declined significantly in the months following the March 2020 lockdown, aligning with implementation of stringent social distancing measures. These findings contribute valuable empirical information into the social behaviour in Norway during the early pandemic, which can be used to enhance policy-relevant models for addressing future crises when mitigation measures might be implemented.

**Supplementary Information:**

The online version contains supplementary material available at 10.1186/s12889-024-18853-8.

## Introduction

During the beginning of 2020, COVID-19 rapidly spread around the globe, resulting in a considerable burden on public health and economic welfare of societies [1]. In order to limit the burden of the disease and prevent a collapse of healthcare services, governments early imposed regulations requiring people to reduce social interactions and other risky behaviours in an effort to contain the transmission of COVID-19 [2, 3].

In Norway, the first COVID-19 case was detected on 26 February [4]. Following a rapid increase in COVID-19 hospitalisations, on 12 March, the Norwegian government issued a national lockdown with strict border control, mandatory home quarantine and isolation in case of exposure and infection, closure of educational institutions and shops except for essential goods and medicine and cancellation of sports and cultural events. In addition, there were recommendations to increase hygiene, work from home if possible, and avoid public transportation and domestic travel [5, 6]. After a subsequent drop in COVID-19 hospitalisations, a gradual reopening started late April 2020, prioritising day-care and primary schools, later followed by high schools, and allowance of public events of limited size [5, 6]. From end of May, a three-tiered infection prevention and control (IPC) system was implemented in primary schools [7, 8]. The guidelines advised the establishment of small cohorts of children and staff with limited interaction between cohorts, alongside hygiene measures, timely testing and isolation of symptomatic cases, and tracing and quarantine of their contacts. In June 2020, bars and sports activities could reopen under 1-metre social distance conditions, and international travel restrictions were scaled back, except for regions with high infection levels. COVID-19 hospitalisations remained low in Norway throughout the summer and until mid-autumn. By late summer 2020, a seroprevalence study suggested that 0.6% (95% confidence intervals (CI): 0.2–1.2%) of the population had been infected with COVID-19 [9].

Mathematical models were key in informing public health policy decision-making during the COVID-19 pandemic. The models provided a framework for informed assessment of the epidemic evolution, short-term forecasting, and estimation of effects of control measures based on transparent assumptions and data. However, the models were constrained by scarce knowledge about the virus, infection prevalence, immune response, uncertainties related to data, including underreporting of cases, and, not least, missing data about social mixing patterns during a period of unprecedented control measures [10]. Due to the dynamic nature of the COVID-19 epidemic, the models needed constant updates to provide timely, data-driven estimates of the effective reproduction number [11, 12] and short-term projections of the health-care burden [13].

SARS-CoV-2 is primarily transmitted via respiratory droplets and close contact routes. The use of quantitative country- and age-specific data on social mixing has been found essential in models studying transmission dynamics of close-contact infections [14–16]. In early 2020, social contact pattern data for Norway were available from a survey conducted in 2017 [17], inspired by the earlier POLYMOD survey [15]. These social mixing data gathered during a period of normalcy without social distancing measures were unlikely to be representative during the pandemic. Instead, the behaviour was expected to change during the rapidly evolving crisis in response to policies, disease incidence and health risk perceptions, and there was an urgent need for updated social mixing data to support the ongoing management of the disease and improve the general understanding of contact patterns of relevance for outbreak preparedness.

Here, we report on a panel study conducted in Norway during the first year of the pandemic, providing novel information about social mixing patterns during the early phase of the pandemic. By comparing the survey results to Norwegian data collected in a 2017 survey, we aimed to quantify changes in the social contacts and mixing patterns and, consequently, their impact on the transmissibility of SARS-CoV-2.

## Methods

### Data collection and recruitment

The Norwegian panel survey was part of the international online CoMix study collecting data on social contact patterns, behaviour and attitudes over the course of the COVID-19 pandemic in European countries [18]. The CoMix study was inspired by the POLYMOD study [15] and was launched in March 2020 in United Kingdom, Belgium and the Netherlands. The Norwegian Institute of Public Health and the University of Bergen joined this collaboration, resulting in a 6 data collection waves for the Norwegian panel survey conducted between April and October 2020. Details on the CoMix study, including the protocol and survey questionnaire have been published previously [19]. The CoMix survey questionnaire was translated into Norwegian.

The market research company Ipsos was responsible for carrying out the survey in Norway and other countries participating in the CoMix study. In Norway, Ipsos recruited a nationally representative adult population sample with respect to age, gender, geographical location, and socio-economic status from an existing online panel [20]. The company developed the web-based form from the translated CoMix questionnaire.

**Fig. 1:**
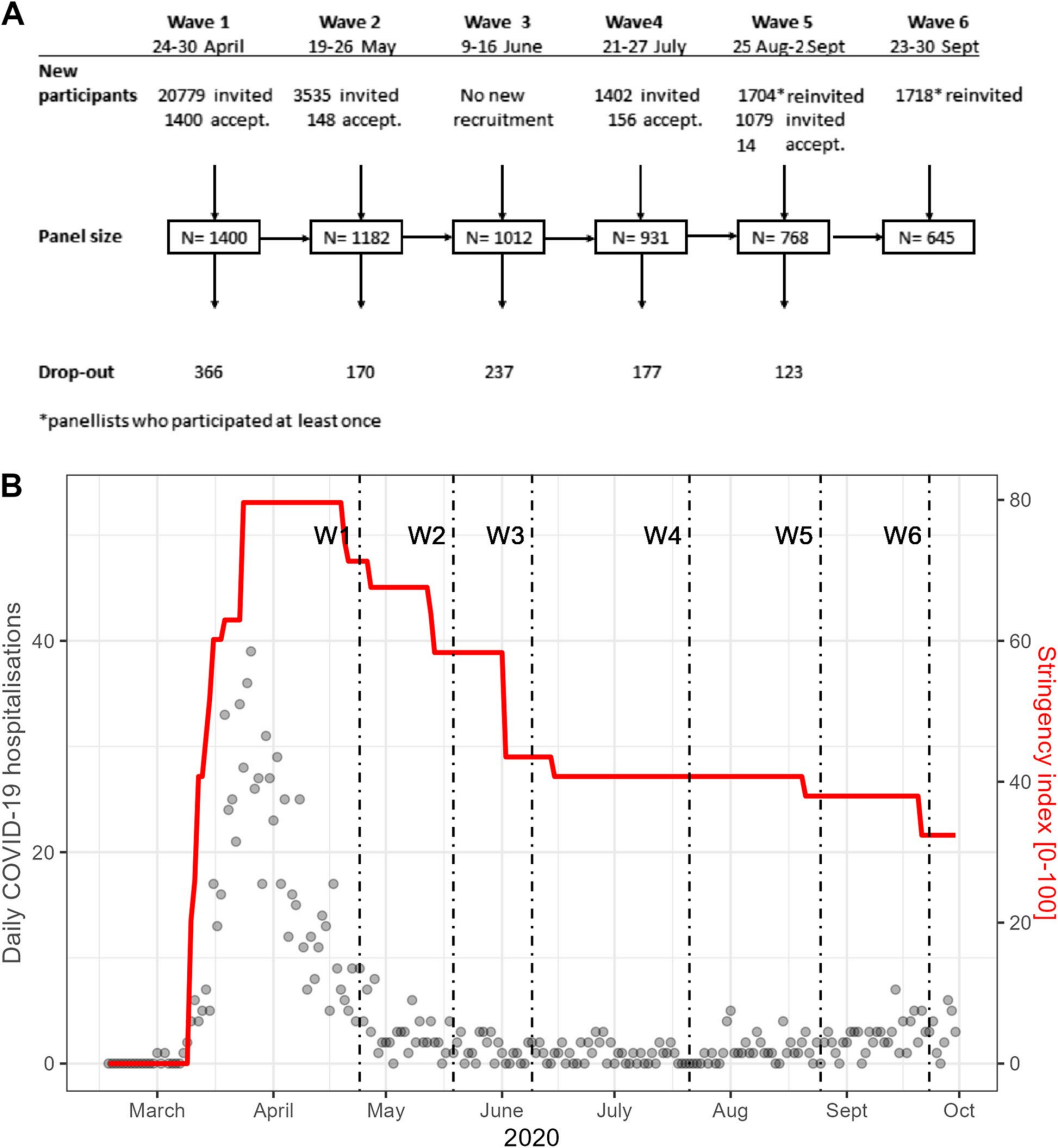
Timeline, Norwegian CoMix survey: **A** Overview of recruitment and data collection, April to September 2020, **B** shows the number of daily hospital admissions in Norway and the stringency index during the survey period, with start dates for each CoMix wave indicated with a vertical line. The stringency index is a composite measure summarising nine response instruments, such as school closures, work-place closures, and travel restrictions, re-scaled to a value of 0 (no interventions) to 100 (strictest response)

In each of the six data collection waves, denoted as CoMix waves, participants were invited by email to register their contacts on a randomly assigned weekday of each week of data collection. In case of non-response, up to two reminder emails were sent. To account for dropouts, Ipsos recruited additional panellists during the study period with similar characteristics in terms of age and gender. Participants received “panellist points” exchangeable for shopping vouchers for each questionnaire they filled out. Figure 1 shows an overview of the Norwegian CoMix survey with dates and numbers of participants and substitutes recruited in the CoMix waves.

Participants provided socio-economic and demographic information during the enrolment survey. Other data were collected using an identical questionnaire in each CoMix wave. The questionnaire included detailed questions about their social contacts, exposure to social distancing measures, uptake of preventive measures, and attitudes and risk perception regarding the COVID-19 pandemic. This article focuses on the social contact data and related personal protection measures (hand washing, use of hand sanitiser and face masks) while analyses of other survey topics have been published previously [20, 21] or are planned to be published soon.

### Social contact data

CoMix survey participants were asked to detail their social contacts on the day preceding the email invitation (24 h). The questionnaire defined a social contact as either an in-person conversation (exchange of at least few words) or physical contact (involving touch, e.g., handshake, embracing, contact sports). Participants could register up to 45 individual contacts per CoMix wave with information about the (estimated) age and gender of the contact, their relations, location of the encounter (home, work, school etc.), duration and frequency of interaction, type of contact (physical or not) and whether the contact occurred outdoors or indoors. In addition, questions on group contacts followed the questions on individual contacts and were added to surveys after CoMix wave 1. Group contacts were added to accommodate participants who could not record details of all individual contacts that they had, e.g., clients, patients, students etc. To address potential biases, uncertainties, and disparities between the CoMix and baseline survey questionnaires, we made the decision to exclude grouped contacts from our current study. There were several reasons behind this choice. Firstly, the first CoMix wave did not gather grouped data, making it impossible to ensure comparability of contact numbers across different data collection waves. Secondly, we lacked information regarding the age distribution of grouped contacts in the 2017 baseline survey. Lastly, we identified instances where a few individuals reported exceptionally high contact numbers, which, in certain cases, were due to typographical errors or misunderstandings. More details on group contacts are presented in supplement, Sect. 1.

### Baseline survey

We compared the Norwegian CoMix data to the Norwegian social contact data collected through a cross-sectional survey (single data collection) conducted by the Norwegian Institute of Public Health in 2017 [17]. Participants were randomly selected from the population registry by Statistics Norway [22] and received invitations and paper questionnaires by mail. Enrolment occurred in late April with a reminder sent to non-responding invitees in September. In the study 4 792 people were invited, of which 2 593 were adults (≥ 18 years). From the invitees, 565 (response rate, 12%) individuals aged 0 to 92 years participated including 309 (response rate, 12%) adults.

Participants were asked to fill in their social contacts on a particular weekday using a paper diary similar to the one used in the POLYMOD study [15]. They could report up to 49 individual contacts, and additional grouped contacts described in free text format (group contacts were excluded as explained above). Because the 2017 Norwegian questionnaire closely resembles the CoMix survey, we used it as baseline measurement of social contacts during the pre-pandemic period in Norway. Henceforth, the study is referred to as the ‘baseline survey’.

### Control measures

In Norway, the stringency of control measures implemented during the pandemic varied depending on the evolving epidemiological situation. We mapped the main social distance measures implemented during the CoMix data collection periods and those are presented in the supplement, Sect. 3. In Fig. 1, we present the stringency index (Government Response Stringency Index) along with the daily number of hospitalisations over the first period of the pandemic in order to get a better understanding of the situation. The Stringency Index is a tool developed by researchers from the Blavatnik School of Government at the University of Oxford to quantify and compare the strictness of government policies implemented in response to the COVID-19 pandemic. It is a composite measure summarizing nine response instruments, such as school closures, work-place closures, and travel restrictions, re-scaled to a value of 0 (no interventions) to 100 (strictest response) (see [23] for a full description).

### Data analysis

We compared the age, gender, and county of residence of our survey participants to the 2020 mid-year estimates of the Norwegian adult population provided by Statistics Norway [22] to assess the representativeness of our study population in the different CoMix waves.

We computed the mean number of daily contacts for each CoMix wave, stratified by participant characteristics, including age group, gender, household size and day of the week. We also present comparative results with the baseline survey as reference. Using the survey design in Stata 16.0, for each CoMix wave, we weighted the analysis by age group and gender to obtain population-representative results. The weighted mean number of contacts were presented with 95% CI. Data on personal protective measures were analysed by calculating percentages of participants reporting hand washing and using hand sanitisers and face masks.

We calculated age-specific contact matrices for the baseline survey, and for the CoMix survey using imputation of contacts in children, categorising age groups into 0–4, 5–17, 18–29, 30–49, 50–69, and 70 + years. These matrices were adjusted according to the population size in Norway in the different age groups, forming population contact matrices *C=(c_ij).* According to the social contact hypothesis, we assumed that the age-specific number of social contacts is proportional to the potential transmission events [14]. In this context, the population contact matrix *C* is related to the next-generation transmission matrix as NG = q*C, where *q* is a scaling factor capturing various aspects, including the infectivity and the susceptibility and implicit scaling of the contacts as they were measured per day, while the duration of infectivity will last longer. Each element of the next-generation matrix, *ng_ij*, represents the anticipated number of new infections in age group *i* caused by an infected individual in age group *j*, under the assumption of a completely susceptible population. The basic reproduction number, R0, is obtained as the largest eigenvalue of this matrix.

To gauge shifts in transmissibility owing to changes in contact patterns, we followed the approach described by Hens et al. [24]. Specifically, we determined the relative change in R0 by calculating the ratios R0_CoMix / R0_baseline = Max eigenvalue (q*C_CoMix) / Max eigenvalue (q*C_baseline). We assumed that the social mixing pattern in February-March 2020 before the implementation of social distancing measures were comparable to those measured in the 2017 survey. Further, we assumed that the duration of the infectious period did not change, that the per-contact transmission probability remained constant and that all age groups had the same per-contact transmission probability given infection. In this case, the scaling factor q is constant and cancels out and the relative change in R0 reduces to determining the ratio between the largest eigenvalues between the CoMix and the baseline contact matrices in 2017.

Given that no data were collected for children under 18 years in the CoMix study, we employed imputation to generate complete population matrices using the method proposed by Klepac et al. [25] previously applied in several studies [19, 26, 27]. This procedure involves calculating the ratio of the maximum eigenvalues between the CoMix matrices and a baseline matrix for age groups present in both surveys (adults) and filling in missing matrix elements in the CoMix matrices by the corresponding child-to-child elements in the baseline matrix multiplied by this eigenvalue ratio. Instead, we imputed child-to-adult contacts from adult-to-child contacts in the CoMix matrix.

To assess the uncertainty in the contact matrices, we bootstrapped by sampling participants with replacements 10,000 matrices from the 2017 baseline study and each CoMix wave. First, we selected the adult-to-adult sub-matrices, adjusting for the reciprocity of contacts between age groups and the mid-year population data in 2017 and 2020 from Statistics Norway [22]. For each baseline-CoMix sample pair, we calculated the R0 ratio of the largest eigenvalues (*max λ* CoMix/*max λ* baseline) and imputed the contacts of children in the CoMix matrix as described above. We repeated the same procedure, adjusting for reciprocity of contacts and calculating the R0 ratio for the full population baseline-imputed CoMix matrix pair.

This entire process was conducted separately for all contacts and physical contacts, and for different locations (home, school, work, and ‘other’). The R0 ratio distributions of adult contacts and imputed contacts are shown in supplement Figure S3 and S4.

Before the lockdown in March 2020, a mathematical model calibrated to Norwegian hospitalisation data estimated an R0 of 2.69 (sd = 0.3) [13, 28]). We multiplied this distribution with the R0 ratio distribution obtained from the imputed population matrices (all contacts and physical contacts) to calculate R0 under the social distancing measures.

The imputed contacts of children are based on adult behavioural changes and therefore subject to uncertainty. For this reason, we conducted a sensitivity analysis to assess the impact of children-to-children, repeating the calculations of all contacts and physical contacts assuming a reduction of 25%, 50%, 65% and 80% compared to the baseline survey contacts rates within the (0–4,5–17) age groups.

Finally, we characterised the social mixing pattern by calculating the disassortativity index, as suggested by Farrington et al. [29]. The index was standardized relative to homogeneous mixing, with a value of 1 indicating such mixing. Values greater than 1 indicated disassortative mixing with a preference for contacts outside one’s own age group, while values less than 1 indicated assortative mixing with a tendency for contacts within one’s own age group.

In the 2017 baseline survey data we performed multivariate imputation by chained equations (*N* = 10 datasets) to account for missing values of physical contacts (10.8%), age of contacts (1.7%) and location (0.4%) using the R-package MICE. When adjusting for age, we used a threshold value of 3 to limit the influence of single participants.

We used Stata version 16 (Stata Corporation, College Station, Texas, US) and R version 4.0.0 with the socialmixr package ver. 0.2.0 and the MICE package ver. 3.15.0 to analyse the data.

## Results

### Sample characteristics

The Norwegian CoMix study included 1 718 participants reporting 5 938 questionnaires with information about a total of 22 074 contacts. In the first CoMix wave, 20 779 adults were invited, and 1 400 participated. Despite further recruitment, the sample size gradually decreased during subsequent data collection waves reaching 645 participants in the final wave (Fig. 1, panel A). Of the original 1,400 participants, 315 took part in all six CoMix waves.

The demographic characteristics of the participants in the CoMix waves and adults in the baseline survey are presented in Table 1. Approximately half of the participants were male, and the median age varied between 49 and 54 years across the CoMix waves, compared to 47 years in the Norwegian adult population. Additionally, the median age showed a slight increase over the study period. The mean household size was 2 with a range from 1 to 12. While panellists from all counties in Norway participated, Oslo was somewhat over-represented in all CoMix waves. Further details regarding the representativeness are provided in supplementary material, Sect. 2.

**Table 1:** Descriptive characteristics in the 2017 baseline survey and in each CoMix wave in 2020. *Starting date of each CoMix data collection wave is indicated in brackets*

Characteristics	Number of participants (%)						
	Baseline survey 2017 *n* = 309	Wave 1 (24 April), *n* = 1400	Wave 2 (19 May), *n* = 1182	Wave 3 (9 June), *n* = 1012	Wave 4 (21 July), *n* = 931	Wave 5 (25 Aug), *n* = 768	Wave 6 (23 Sept), *n* = 645
**Gender**							
Male	146 (47%)	702 (50%)	610 (52%)	534 (53%)	487 (52%)	421 (55%)	357 (55%)
Female	163 (53%)	698 (50%)	569 (48%)	475 (47%)	441 (47%)	345 (45%)	287 (44%)
Missing	0 (0%)	0 (0%)	3 (0%)	3 (0%)	3 (0%)	2 (0%)	12 (1%)
**Age group**							
18–29	46 (15%)	217 (16%)	121 (10%)	98 (10%)	78 (8%)	57 (7%)	70 (11%)
30–49	36 (12%)	509 (36%)	410 (35%)	352 (35%)	300 (32%)	257 (34%)	210 (33%)
50–69	93 (30%)	493 (35%)	458 (39%)	406 (40%)	406 (44%)	319 (42%)	262 (41%)
70+	134 (43%)	181 (13%)	193 (16%)	156 (15%)	147 (16%)	135 (18%)	103 (16%)
**Day of week**							
Weekday	221 (72%)	1003 (72%)	955 (81%	930 (82%)	782 (84%)	584 (76%)	630 (98%)
Weekend	87 (28%)	397 (28%)	227 (19%)	82 (18%)	149 (16%)	184 (24%)	15 (2%)
Missing	1 (0%)	0 (0%)	0 (0%)	0 (0%)	0 (0%)	0 (0%)	0 (0%)
**Household size**							
1	64 (21%)	320 (23%)	300 (25%)	278 (27%)	269 (29%)	200 (26%)	179 (28%)
2	169 (55%)	453 (32%)	458 (39%)	387 (38%)	358 (38%)	315 (41%)	251 (39%)
3–4	53 (17%)	482 (35%)	333 (28%)	275 (27%)	258 (28%)	216 (28%)	177 (27%)
5+	16 (5%)	145 (10%)	91 (8%)	72 (7%)	46 (5%)	37 (5%)	38 (6%)
Missing	7 (2%)	0 (0%)	0 (0%)	0 (0%)	0 (0%)	0 (0%)	0 (0%)
**Education**							
Nursery, primary school	32 (10%)	57 (4%)	42 (4%)	39 (4%)	41 (5%)	28 (4%)	26 (4%)
Secondary school	121 (39%)	349 (25%)	282 (24%)	232 (23%)	216 (23%)	186 (24%)	153 (24%)
Higher education^b^	151 (49%)	994 (71%)	858 (72%)	741 (73%)	674 (72%)	554 (72%)	466 (72%)
Missing or Other	5 (2%)	0 (0%)	0 (0%)	0 (0%)	0 (0%)	0 (0%)	0 (0%)
**Daily activity**							
At home^a^	188 (61%)	437 (31%)	416 (35%)	356 (35%)	371 (40%)	392 (51%)	238 (37%)
School/ education	28 (9%)	110 (8%)	63 (5%)	49 (5%)	34 (4%)	355 (46%)	35 (5%)
Employed	77 (25%)	853 (61%)	703 (60%)	607 (60%)	526 (57%)	21 (3%)	372 (58%)
Missing or Other	16 (3%)	0 (0%)	0 (0%)	0 (0%)	0 (0%)	0 (0%)	0 (0%)
**High risk group**^c^							
No	NA	791 (57%)	641 (54%)	560 (55%)	472 (51%)	392 (51%)	351 (54%)
Yes	NA	561 (40%)	510 (43%)	425 (42%)	433 (46%)	355 (46%)	275 (43%)
Do not know/ do not wish to answer	NA	48 (3%)	31 (3%)	27 (3%)	26 (3%)	21 (3%)	19 (3%)
**Hand hygiene (washed hands/ used sanitizer)**^d^							
No	NA	63 (5%)	65 (6%)	58 (6%)	44 (5%)	39 (5%)	24 (4%)
Yes	NA	1 337 (95%)	1 117 (94%)	954 (94%)	887 (95%)	729 (95%)	621 (96%)
**Used Face Mask**^d^							
No	NA	1 339 (96%)	1 135 (96%)	981 (97%)	907 (97%)	719 (94%)	585 (91%)
Yes	NA	61 (4%)	47 (4%)	31 (3%)	24 (3%)	49 (6%)	60 (9%)

*NA *Not available

^a^In this category we have included people who reported they have retired, were unable to work

^b^As higher education, we have grouped together people that had “Occupational training/Technical school”, “Higher education < 4 years” and “Higher education ≥ 4 years”

^c^Risk groups were defined as the one that are recommended to be vaccinated for influenza in United Kingdom. https://www.nhs.uk/conditions/vaccinations/who-should-have-flu-vaccine/. Defined on the question “Are you in a high-risk group under which the annual influenza vaccine would usually be offered?”. Originally 516 participants reported that they had high risk of COVID-19 infection or complications, but after assessing the available information (including pregnant women and people above 65 years old) we assigned 561 (40%) to belong in a risk group. Overall, few pregnant women participated in the Comix study ranging from 4 to 19 over the six waves (around 1% per wave)

^d^In the CoMix survey, participants reported whether they had at least once washed their hands with soap or used hand sanitiser during the last three hours prior to the survey. Regarding the use of face mask, participants were asked whether they used a face mask during the day prior to the survey day

### Drop in the mean number of contacts

In the baseline survey, 2.3% of the adult participants reported zero contacts on the survey day, while in the CoMix study this was 8% (533/5 938) overall. This number increased from 6% in April 2020 to 9–12% in the following data collection waves.

**Fig. 2:**
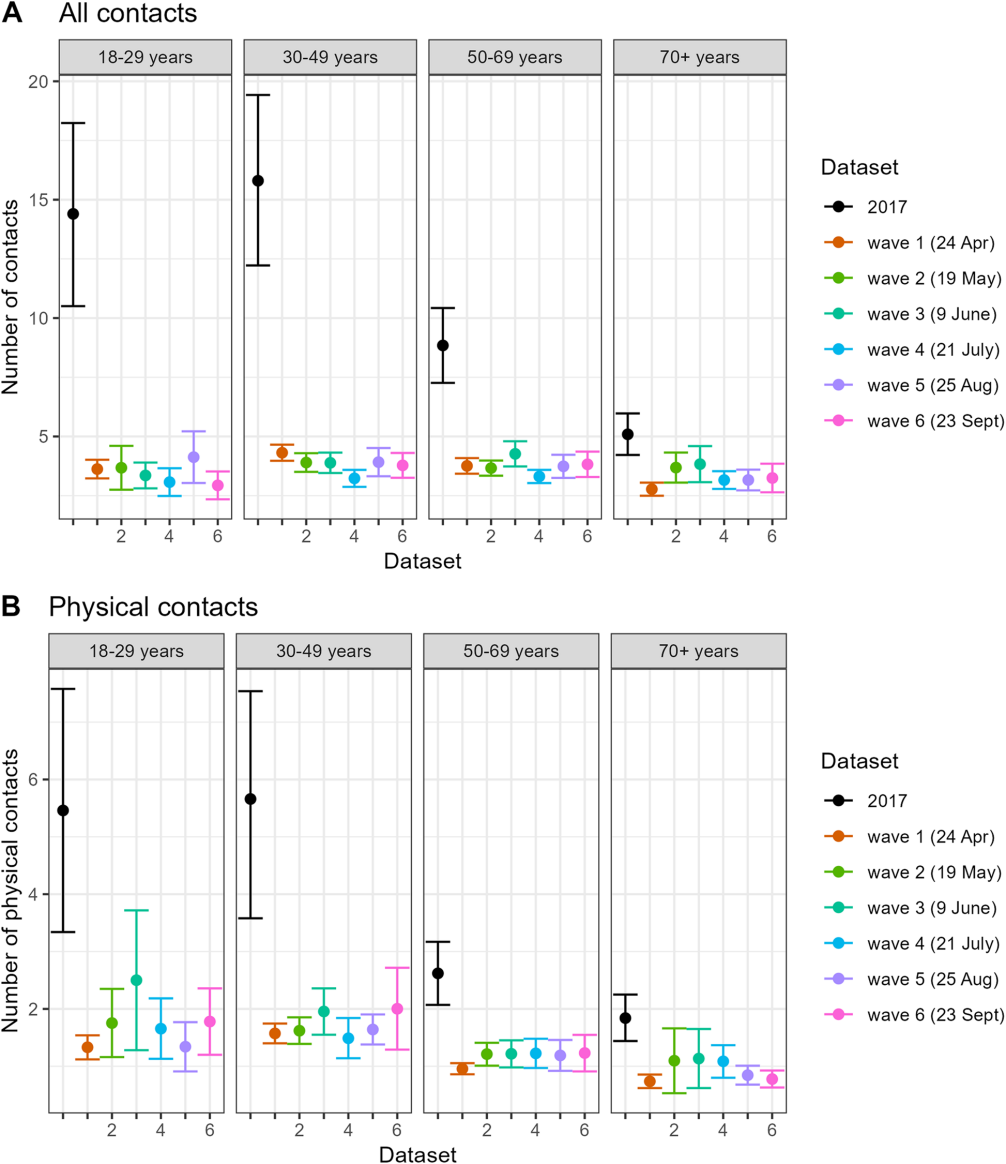
Mean number of daily contacts for the 2017 baseline survey and the CoMix waves: Mean number of daily contacts for the 2017 baseline survey and the CoMix waves (starting date of each CoMix data collection wave is indicated in brackets), with 95% confidence intervals, stratified by participant age group: **A** for all contacts and **B** for physical contacts reported. These results are weighted by gender

The mean number of daily contacts remained low and stable between April and September, varying from 3.2 (95% CI 3.0 − 3.4) in July (CoMix wave 4) to 3.9 (95% CI 3.6–4.2) in June (CoMix wave 3) among adults (Table 2). In comparison, in the baseline survey the mean number of contacts among the adults was 11.9 (95% CI 10.2–13.5), indicating a striking 67–73% decline in social contacts. The largest drop was found in younger adults aged 18–49 years, while in people aged 70 years and older the number was affected least (Fig. 2A).

**Table 2:** Mean number of daily contacts: Summary of the mean number of daily contacts reported by adult participants (≥ 18 years) in the 2017 baseline survey and in each CoMix wave in 2020 (starting date of each CoMix data collection wave is indicated in brackets). These results are weighted by gender and age group

Characteristic	Value	Mean number of contacts (95% Confidence Intervals)						
		Baseline survey 2017, *n* = 309	Wave 1 (24 April), *n* = 1400	Wave 2 (19 May), *n* = 1182	Wave 3 (9 June), *n* = 1012	Wave 4 (21 July), *n* = 931	Wave 5 (25 Aug), *n* = 768	Wave 6 (23 Sept), *n* = 645
Overall Contacts		11.9 (10.2–13.5)	3.8 (3.6-4.0)	3.8 (3.5–4.2)	3.9 (3.6–4.2)	3.2 (3.0-3.4)	3.8 (3.5–4.2)	3.5 (3.3–3.8)
**Physical Contacts**^a^		4.1 (3.2-5.0)	1.2 (1.1–1.3))	1.4 (1.3–1.6)	1.7 (1.4-2.0)	1.4 (1.2–1.6)	1.3 (1.2–1.5)	1.5 (1.2–1.8)
**Gender**	Male	11.1 (8.7–13.5)	3.7 (3.4–3.9)	3.8 (3.2–4.4)	3.5 (3.2–3.7)	3.0 (2.7–3.2)	3.5 (3.0-3.9)	3.3 (2.9–3.7)
	Female	12.7 (10.4–14.9)	3.9 (3.6 4.1)	3.8 (3.5–4.2)	4.4 (3.9–4.9)	3.5 (3.2–3.8)	4.2 (3.6–4.7)	3.8 (3.4–4.2)
**Age group**	18–29	14.4 (10.5–18.2)	3.6 (3.2-4.0)	3.7 (2.8–4.6)	3.4 (2.8–3.9)	3.1 (2.5–3.7)	4.1 (3.0-5.2)	2.9 (2.3–3.5)
	30–49	15.8 (12.2–19.4)	4.3 (4.0-4.7)	3.9 (3.5–4.3)	3.9 (3.5–4.3)	3.2 (2.9–3.6)	3.9 (3.3–4.5)	3.8 (3.3–4.3)
	50–69	8.8 (7.3–10.4)	3.8 (3.4–4.1)	3.7 (3.3-4.0)	4.3 (3.7–4.8)	3.3 (3.0-3.6)	3.7 (3.3–4.2)	3.8 (3.3–4.4)
	70+	5.1 (4.2-6.0)	2.8 (2.5–3.1)	3.7 (3.1–4.3)	3.8 (3.1–4.6)	3.2 (2.8–3.5)	3.2 (2.7–3.6)	3.2 (2.6–3.9)
**Household size**	1	11.0 (6.2–15.9)	2.6 (2.3–2.9)	2.7 (2.2–3.2)	3.2 (2.7–3.7)	1.9 (1.6–2.2)	2.9 (2.2–3.6)	2.9 (2.3–3.5)
	2	7.9 (6.5–9.3)	3.4 (3.1–3.7)	3.2 (2.9–3.4)	3.6 (3.2-4.0)	3.2 (2.9–3.5)	3.3 (2.9–3.7)	3.5 (3.0-3.9)
	3+	16.6 (13.6–19.5)	4.6 (4.4–4.9)	4.9 (4.4–5.5)	4.6 (4.2–5.1)	4.2 (3.9–4.6)	4.9 (4.2–5.5)	4.1 (3.6–4.6)
**Daily Activity**	At home^b^	7.0 (5.6–8.3)	2.8 (2.6-3.0)	3.2 (2.8–3.5)	3.2 (2.8–3.6)	2.8 (2.6-3.0)	3.0 (2.7–3.3)	2.8 (2.4–3.2)
	School/ education	17.7 (12.8–22.7)	3.2 (2.7–3.7)	4.7 (2.8–6.6)	2.8 (2.0-3.5)	2.9 (2.0-3.8)	4.2 (2.6–5.7)	2.7 (1.9–3.5)
	Employed	14.3 (11.6–17.0)	4.4 (4.1–4.7)	3.9 (3.7–4.2)	4.4 (4.0-4.8)	3.5 (3.2–3.8)	4.2 (3.7–4.7)	4.1 (3.7–4.5)
**Weekend**	No	12.6 (10.6–14.7)	3.5 (3.2–3.8)	4.4 (3.1–5.8)	4.1 (3.3–4.9)	2.6 (2.2-3.0)	2.7 (2.3-3.0)	4.0 (1.9-6.0)
	Yes	10.3 (7.8–12.9)	3.9 (3.7–4.1)	3.7 (3.4–3.9)	3.9 (3.6–4.2)	3.3 (3.1–3.6)	4.1 (3.7–4.5)	3.53 (3.2–3.8)
**Risk group**^c^	No	NA	4.2 (3.9–4.4)	3.9 (3.4–4.4)	4.0 (3.6–4.4)	3.2 (3.0-3.5)	4.2 (3.7–4.8)	3.7 (3.3–4.1)
	Yes	NA	3.3 (3.1–3.5)	3.7 (3.3–4.1)	3.9 (3.6–4.4)	3.2 (2.9–3.4)	3.4 (3.0-3.8)	3.4 (3.0-3.8)
**Norwegian-born**	No	15.9 (8.6–23.1)	3.7 (3.0-4.5)	3.2 (2.5–3.9)	3.2 (2.5-4.0)	3.2 (2.3–4.1)	4.1 (2.3-6.0)	3.1 (2.1–4.1)
	Yes	11.5 (9.9–13.1)	3.8 (3.6-4.0)	3.9 (3.5–4.3)	4.0 (3.7–4.4)	3.2 (3.0-3.4)	3.8 (3.5–4.3)	3.8 (3.5–4.2)
**County**	Agder	NA	3.7 (3.1–4.4)	4.2 (3.3–5.1)	4.4 (3.1–5.6)	3.4 (2.6–4.2)	3.5 (2.8–4.2)	3.6 (2.1–5.1)
	Innlandet	NA	3.4 (2.4–4.5)	3.3 (2.3–4.3)	3.3 (2.2–4.4)	2.9 (2.2–3.7)	3.5 (1.5–5.5)	3.8 (1.9–5.6)
	Møre og Romsdal	NA	4.1 (3.2–5.1)	5.1 (2.4–7.8)	4.8 (3.3–6.3)	3.4 (2.7-4.0)	3.6 (2.65–4.5)	4.0 (2.7–5.2)
	Nordland	NA	4.0 (2.8–5.2)	3.9 (2.9–4.9)	5.3 (2.8–7.8)	4.4 (2.9–5.9)	4.1 (2.53–5.6)	4.5 (3.1-6.0)
	Oslo	NA	3.4 (3.0-3.8)	3.8 (2.4–5.2)	3.1 (2.7–3.5)	2.8 (2.4–3.2)	3.4 (2.69–4.2)	3.2 (2.5–3.8)
	Rogaland	NA	4.4 (3.7–5.2)	3.9 (3.1–4.6)	3.8 (2.9–4.8)	3.2 (2.6–3.9)	3.6 (2.77–4.5)	3.1 (2.5–3.7)
	Troms og Finnmark	NA	4.4 (3.4–5.5)	4.0 (2.6–5.4)	4.6 (3.2–5.9)	3.8 (2.6–4.9)	4.1 (2.81–5.3)	3.9 (2.8–4.9)
	Trøndelag	NA	3.6 (3.1–4.1)	3.9 (3.2–4.7)	4.0 (3.2–4.7)	3.6 (2.7–4.5)	4.0 (2.57–5.5)	4.5 (3.1–5.9)
	Vestfold og Telemark	NA	4.0 (3.5–4.5)	4.5 (3.6–5.5)	4.2 (3.3–5.1)	3.2 (2.7–3.8)	4.7 (3.24–6.1)	3.5 (2.7–4.2)
	Vestland	NA	4.1 (3.5–4.7)	3.3 (2.8–3.8)	4.2 (3.3–5.1)	3.3 (2.7–3.9)	4.5 (3.14–5.9)	3.6 (2.7–4.6)
	Viken	NA	3.5 (3.1–3.8)	3.5 (3.2–3.9)	4.0 (3.2–4.8)	3.1 (2.7–3.5)	3.6 (2.82–4.4)	3.3 (2.7–3.9)
**Population density (inhabitants/km**^**2**^**)**	≤ 199	NA	4.0 (3.7–4.3)	3.8 (3.5–4.2)	4.4 (3.8–4.9)	3.4 (3.1–3.7)	3.8 (3.3–4.2)	3.7 (3.2–4.1)
	200–999	NA	3.6 (3.4–3.9)	3.9 (3.5–4.4)	3.9 (3.5–4.3)	3.2 (2.9–3.6)	4.0 (3.3–4.6)	3.8 (3.2–4.3)
	≥ 1000	NA	3.5 (3.1–3.9)	3.7 (2.5-5.0)	3.2 (2.8–3.6)	2.9 (2.5–3.3)	3.45 (2.8–4.1)	3.1 (2.5–3.6)
**Indoors**^d^		NA	3.1 (2.9–3.2)	3.1 (2.8–3.3)	3.3 (3.0-3.5)	2.8 (2.6-3.0)	3.3 (3.0-3.6)	3.3 (3.0-3.6)
**Outdoors**^d^		NA	1.6 (1.5–1.7)	1.6 (1.5–1.7)	1.5 (1.4–1.7)	1.4 (1.2–1.5)	1.4 (1.2–1.6)	0.8 (0.7-1.0)

*NA *Not available

^a^Some reported contacts did not have the information whether the contact was physical or not filled out. The mean number of contacts here did not take into account missing values

^b^In this category we have grouped people who reported that (i) have retired, (ii) were unable to work and (iii) are/stay at home

^c^Risk groups were defined as the one that are recommended to be vaccinated for influenza in United Kingdom. https://www.nhs.uk/conditions/vaccinations/who-should-have-flu-vaccine/. Defined on the question “Are you in a high-risk group under which the annual influenza vaccine would usually be offered?”. Originally 516 participants reported that they had high risk of COVID-19 infection or complications, but after assessing the available information (including pregnant women and people above 65 years old) we assigned 561 (40%) to belong in a risk group

^d^A contact could have been made in more than one place at the same day (both outdoors and indoors). Therefore, some contacts have been calculated in both places

The proportion of contacts involving touch (physical) among adults declined during the early pandemic compared to pre-pandemic levels, fluctuating between 28 and 35% over the CoMix waves vs. 38% in the baseline survey. For physical contacts, the mean number was lowest in April (1.2 per day, 95% CI 1.1–1.3), and highest in June (1.7 per day, 95% CI 1.4- 2.0), corresponding to a drop of 73% and 62%, respectively. The age-specific changes were most prominent in younger adults than other adults, similar to that of all contacts (including physical and non-physical) (Fig. 2B).

CoMix participants reported significantly fewer contacts in July, coinciding with the typical summer holiday period in Norway. Aside from this observation, there were few significant differences between the CoMix waves. This is noteworthy, considering that social distance measures were relaxed during the late April-June period (see supplement, Sect. 3 for the timeline of control measures) and the low incidence of COVID-19 infections during the CoMix survey (Fig. 1B, supplement Table S3). However, contacts among adult students (daily activity: school/education) that were low before the summer due to the closure of educational institutions, increased in August (when schools opened) and later decreased towards September as in person teaching was changed to digital teaching.

Persistent differences in contacts between weekdays and weekends were not observed. However, panellists reported fewer contacts during weekdays than weekends (Saturday-Sunday) in July-August (Table 2). Contacts made at home primarily reflected the household size, and the relative reduction of contacts in the summer holiday period (CoMix wave 4) was most pronounced for the smallest households. Indoor contacts constituted a significant proportion, accounting for 65% of all contacts reported in April and rising to 79% by the end of September (supplement, Figure S1; Table S4). In terms of participant characteristics, employed participants reported significantly more contacts than participants with “at home” status across all CoMix waves. The results were more mixed for participants under education (Table 2). There was no significant association found between participant gender, Norwegian-born status or geographic location and the reported number of contacts (Table 2).

### Changes in mixing pattern

**Fig. 3:**
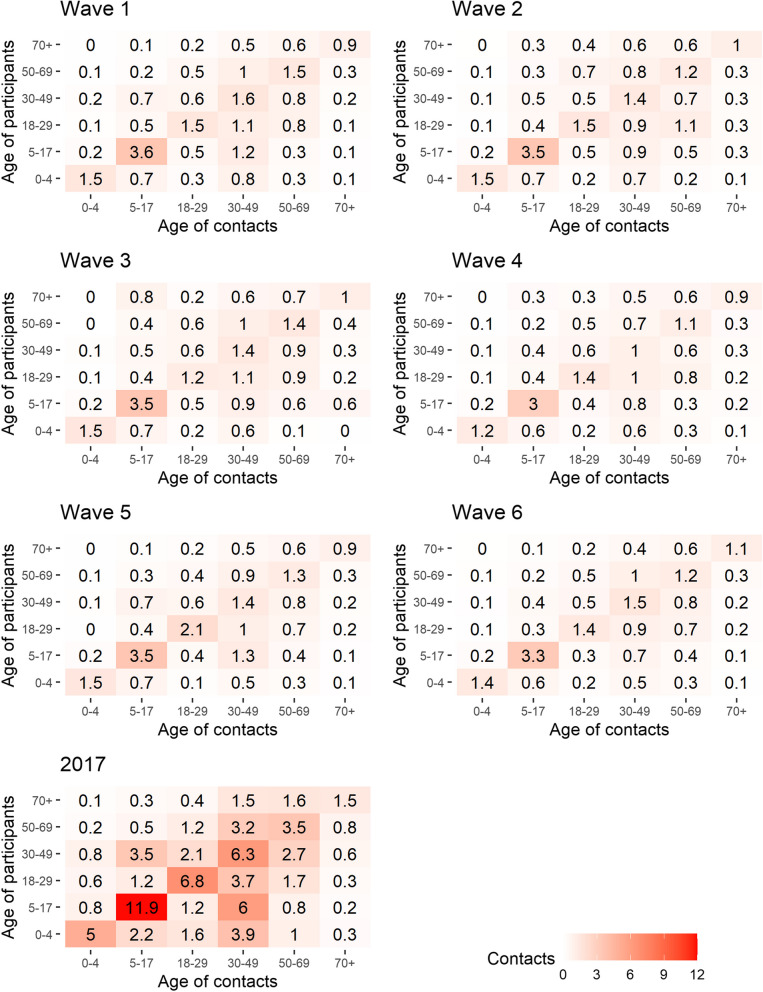
Imputed social contact matrices showing the mean daily number of contacts in the six CoMix waves. For comparison, the corresponding matrix from the 2017 baseline survey is shown below. The matrices represent bootstrap mean values of *N*=10 000 samples. Data were weighted on age and adjusted for reciprocity of contacts

**Fig. 4:**
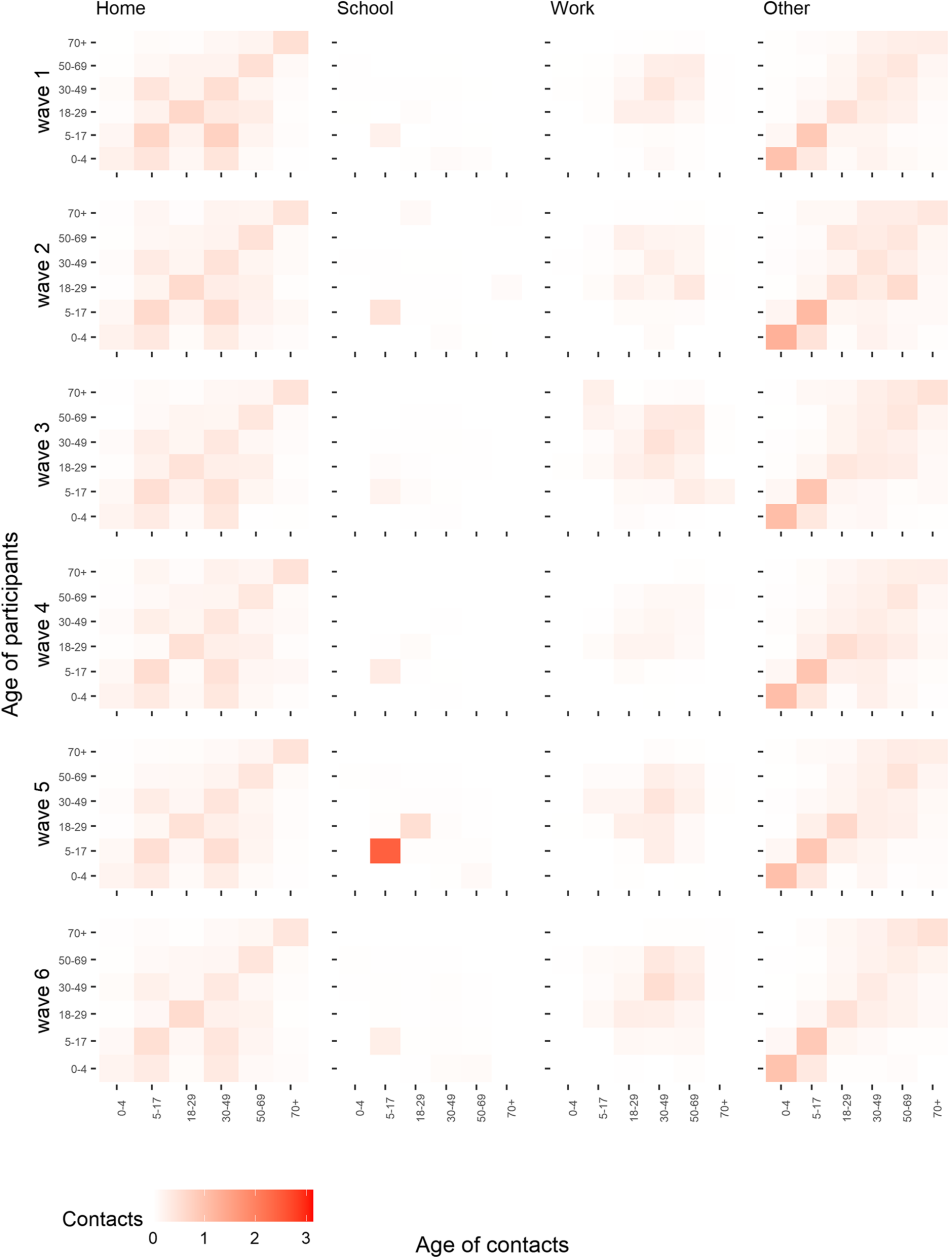
Imputed setting-specific social contact matrices; mean number of daily contacts for the six CoMix waves. Locations include all contacts made in the home, at schools, at workplaces and other community contacts (transport, sport activities etc.). Note that some contacts overlap as multiple settings could be registered for the same contact. The matrices represent bootstrap mean values of *N*=10 000 samples. Data were weighted on age and adjusted for reciprocity of contacts

**Fig. 5:**
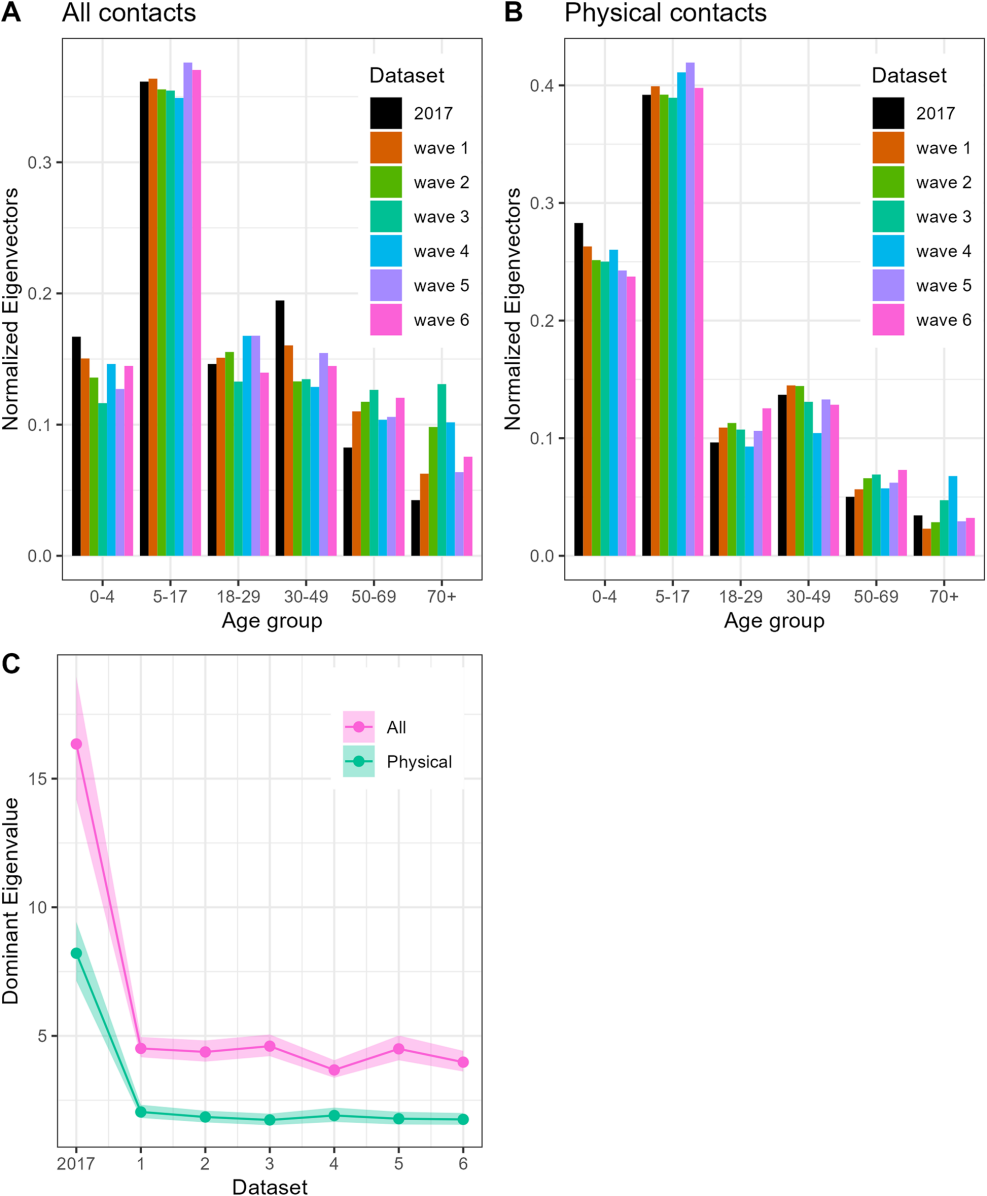
Comparison of CoMix and baseline dominant eigenvectors and eigenvalues: **A** Dominant normalised eigenvectors from contact matrices of all contacts by age group, **B** Dominant normalised eigenvectors from contact matrices of physical contacts by age group, **C** dominant (maximum) eigenvalues of contact matrices for all contacts (red) and physical contacts (green)

The imputed CoMix contact matrices exhibit a general decline in contact rates when compared to the baseline. At the same time the mixing patterns observed in the baseline survey were maintained, characterised by high density along the diagonal (indicating mixing within the same age groups) and high-density “wings” (off-diagonals, representing mixing between children and their parents) (Fig. 3). The location-specific matrices reveal a large degree of consistency across the CoMix waves. An exception is school contact rates, which remained consistently low, except for CoMix wave 5 in late August (Fig. 4). Note, however, that these results should be interpreted with caution. The change is driven by an increase in reported school contacts of adults, leading to higher imputed values in children. Home contacts show mixing primarily with partners of the same age and children, and “other” community contacts display assortative mixing, particularly among the youngest age groups. The dominant eigenvectors of the CoMix matrices, which represents the contribution of different age groups to overall transmission, reveal a slightly more age-homogeneous contribution compared to the pre-pandemic matrix. In both surveys, assuming that behavioural changes in adults is representative of changes in child-related contacts, school-age children made the most significant contribution when considering all contacts (see Fig. 5A), and for physical contacts also the youngest children contribute significantly (Fig. 5B). The mean effective daily contact number (maximum eigenvalue) ranged from 3.7 to 4.6 in the CoMix waves, compared to 16.3 in the baseline survey (Fig. 5C). The sensitivity analysis reducing child-to-child contacts by 25–80% in each CoMix survey wave, compared to 72–77% in the main analysis, indicates a major contribution of children (supplement, Figure S9).

Overall, the imputed CoMix contact matrices of physical contacts (supplement, Figure S5) were rather stable. These contacts were dominated by contacts in the home and ‘other’ settings (supplement, Figure S8), while contacts at school and workplaces were almost absent. The strong influence of household contacts is visible in the dominant eigenvectors where children (0–18 years) and their parents (30–50 years) were primary contributors to potential transmission, aligning with the baseline study (Fig. 5B). The mean effective daily physical contact number varied between 1.7 and 2 throughout the survey, compared to 8.2 in the baseline (Fig. 5C).

The index of disassortativity for all contacts was higher in the CoMix contact matrices, varying between 0.46 and 0.59, with the largest value in July, compared to 0.43 in the baseline survey. This suggests assortative mixing and a slight shift towards more intergenerational mixing during the early stages of the pandemic (supplement, Table S5). Regarding physical contacts, the disassortativity index varied from 0.46 to 0.54, compared to 0.5 in the baseline, indicating minimal change.

### Community contacts

Overall, the largest eigenvalue ratios of the adult population contact matrices categorised by type of contact and location showed a substantial reduction (supplement, Figure S3). The mean ratios of school contacts in adult students were consistently below 0.1, except in August when it reached 0.3. Work contact ratios displayed skewed distributions, but with mean levels typically around 0.2–0.3, except in July (CoMix wave 4), where they dropped to 0.1. These contacts, together with “other” contacts made the largest share of community contacts. Within the category of “other”, physical contacts peaked in July (CoMix wave 4), elevated by encounters associated with vacation activities, particularly contacts in other households, restaurants and bars, and other outside locations (supplement, Table S5). Among adults, contacts involving touch at school and workplaces remained low despite increasing contact numbers in August-September, indicating awareness and adherence to distancing recommendations. The location-specific reductions in the full population matrices with imputed contacts for ages 0–17 years display a similar pattern (supplement, Figure S4). However, these values should be interpreted with caution, as the CoMix study only included adults.

### Estimated R0 reproduction number

**Fig. 6:**
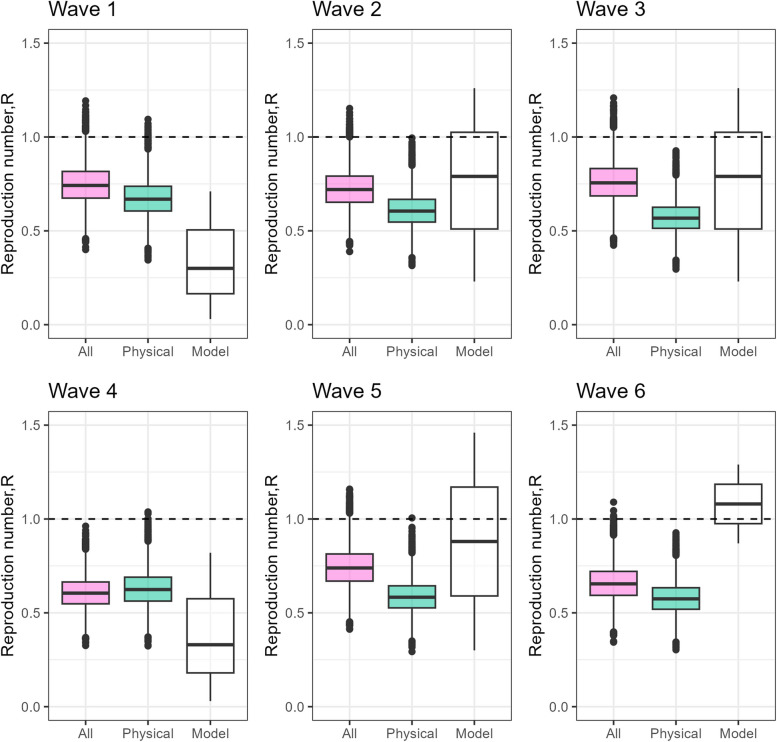
Reproduction numbers: Boxplots showing the estimated basic reproduction numbers, R0, derived from the six CoMix wave surveys. The R0 values were calculated based on contact matrices that consider all contacts (red) and exclusively physical contacts (green). The estimation involves multiplying the maximum eigenvalue ratios (Imp CoMix/baseline) by an initial R0-value established for Norway before the March 2020 lockdown. Note: The eigenvalue ratio, under the assumption of the “social contact hypothesis,” serves as an indicator of the R0-ratio, reflecting how changes in social behavior during the early pandemic influence transmissibility. For additional context, the figure includes white-boxed plots representing the estimated effective reproduction numbers, R_eff, from a Norwegian mathematical model [13, 28], corresponding to the time points of CoMix survey data collection

Figure 6 illustrates the estimated R0 reproduction numbers by considering changing pandemic contact levels compared to pre-pandemic levels using the imputed CoMix contact matrices for all contacts and physical contacts. For comparison, these estimates are presented alongside estimated values of the effective reproduction number from a calibrated model using Norwegian daily admission and test data around the survey collection dates [13, 28]. The R0 numbers based on overall contact matrices exhibited more variability across the CoMix waves compared to the ones based on physical contact matrices and showed a somewhat closer alignment with the model-derived estimates, including a dip in July and increasing transmission in August. Results from the sensitivity analysis, varying the reductions in child-to-child contacts, suggest a reduction in excess of 50% in order for the R0 value to be less than 1, corresponding to a drop in incidence (supplement, Figure S10).

However, both methods (based on overall or physical contact matrices) suggest potential overestimation in April and underestimation in September when compared to the real-time model-derived estimates.

### Personal protective behaviour

Use of personal protective measures, including hand washing, use of hand sanitiser or face masks can impact transmissibility. Hand hygiene in Norway has been strongly recommended since the start of the pandemic (see supplement, Table S3). In our survey, 95% of the participants reported that they had at least once washed their hands or used hand sanitiser in the last three hours, and this was a consistent finding over all six CoMix waves (range 94–96%). Only 3–4% of the participants in April-July reported using face masks, and this number increased to 6% and 9% in August and September, respectively. As a preventive measure for COVID-19, face masks were recommended from mid-August 2020 in the capital Oslo and the surrounding Østfold region during public transportation where a minimum of one-meter distance could not be met [6, 28].

## Discussion

This study provides insights into the changes in social behaviour during the early COVID-19 pandemic in Norway. The CoMix survey started in April 2020, just two weeks after the end of the national lockdown that involved strict social distance measures, including the closure of schools, kindergartens, fitness centres, restaurants, hair salons, etc. While the measures gradually eased towards the summer, certain restrictions remained in place until the study finished in late September [5, 6].

We found a significant reduction of 67–73% in the mean number of reported daily contacts in adults from April to September 2020 compared to the pre-pandemic baseline survey. This reduction was most pronounced among younger adults (< 50 years). The primary factor contributing to the decline was a decrease in community contacts, with contacts within households accounting for the majority of the contacts reported. Our findings are comparable to reductions in contacts reported from other countries in that period. In United Kingdom, a 79% decrease of contacts was reported during lockdowns, lowering to 57% in the summer when measures eased [30]. In China, where interventions were stricter, 86% and 88% reductions were observed in February in Wuhan and Shanghai, respectively [31]. In other countries with data until the autumn of 2020, the mean number of contacts increased significantly during the period (only small increase observed in Norway) following the lockdown or easing of social distance measures, including Belgium (69–80%), the Netherlands (41–72%), Luxemburg (59–82%), and the USA (60–82%) [27, 32–34] but still remained below the pre-pandemic levels.

Interestingly, the Norwegian results showed no noticeable changes in social mixing patterns in response to the scale-back of restrictions, though, certain mitigation measures were maintained. This finding may be attributed to the high level of awareness in the Norwegian population and a general trust in authorities [35]. During this period, the Ministry of Health together with the leadership of the Norwegian Institute of Public Health and the Norwegian Directorate of Health held televised press briefings one to three times per week to communicate restrictions and provide information about preventive measures [5]. The low proportion of reported physical contacts among adults at schools (high schools, technical schools, universities etc.) and workplaces throughout the survey suggests that employers and institutions effectively implemented measures. We estimated the effective mean number of daily contacts per person for the whole population to be around 4–5, and 2–3 for physical contact, corresponding to a drop of 69–78% in expected transmissibility compared to regular social interactions. In the baseline survey, mixing patterns during the early pandemic displayed assortative tendencies that changed somewhat onwards more inter-age group contacts. This shift was driven by a large drop in school contacts in adults, work-related and leisure activity contacts, while contacts within households, which were more age-disassortative, declined less. Connections in the community were primarily in other premises, and these random contacts could potentially support connectivity between population groups (“small-world effects”).

Regarding the estimation of reproduction numbers, our results (assuming similar reduction in children contacts as observed among adults) were broadly consistent with those of an independent model calibrated to Norwegian data [13]. We found that using overall contact matrices as opposed to physical contact matrices gave higher level of consistency with the epidemiological dynamics. Furthermore, the physical contact matrices suggest an initial epidemic driven by children, akin to influenza, while a Norwegian serological survey from January 2021 found no differences in seroprevalence by age group [36].

The Norwegian CoMix study was used to gauge contact contributions from different settings in relation to parameterisation of individual-based models used in scenario analyses and planning purposes during the response to the pandemic. Several other countries also started collecting contact pattern data early in the pandemic including the CoMix partners. The SOCRATES platform brings together the CoMix social contact data from over 20 European countries collected at different points in time throughout the COVID-19 pandemic [37]. A comparison of the contact pattern data among countries has been published elsewhere highlighting the importance of these data in evaluating a diverse range of physical distancing control measures [18].

## Limitations

The low response rate in the online CoMix survey may have resulted in selection bias concerning behavioural characteristics, computer literacy, adherence to mitigation measures, and more. For example, Ipsos obtained only a 7% recruitment rate in CoMix wave 1. Despite anonymity, participants might have felt pressure to report fewer contacts due to the recommendations and restrictions that were in place, which was likely less the case during the baseline survey. Additionally, systematic differences in sample populations and answers between the CoMix and baseline surveys may result from differences in design, recruitment methods, and type of media used for the questionnaire. While the gender balance was well-maintained in both surveys, there were clear differences in age profiles. For example, the baseline survey was more biased towards elderly, with 43% compared being older than 70 years compared to 16% in the CoMix survey. We believe the CoMix survey is fairly representative of a Norwegian adult population, but it may not be optimal for all aspects of comparative analyses with the baseline survey. We should note that the baseline survey had as well a low response rate of 12% also indicating the difficulties of conducting such studies that entail a certain burden on participants. One potential reason for the lower response rate in the CoMix study compared to the baseline might be the extended length of the CoMix questionnaire, which encompassed inquiries about exposure to social distancing measures, uptake of preventive measures, and attitudes and risk perceptions concerning the COVID-19 pandemic.

Another important limitation is the exclusion of children below 18 years in the CoMix survey to expedite the launch and avoid a prolonged process for ethical approval. Children constitute a significant portion of the population with distinct social behaviours. In this study, we addressed the absence of data on children’s contacts by imputation, re-scaling portion of contact matrices from the baseline study based on behavioural changes in adults. This procedure is potentially problematic because it assumes that children and adults respond similarly. Additionally, some control measures during the time of the study were designed to protect children’s usual activities (school remained open with IPC measures, supplement Table S3) more than adults. Although we have gauged children-to-adult contacts using the available information on adult-to-children contacts, it does not remedy the lack of data on children-to-children contacts.

Another approach to address the missing data on children contacts involves the use of data on contacts of children from other countries that are available in later periods of the pandemic. A cross-country analysis of the CoMix study 2020–2022 reported a median number of daily contacts of 9.8 and 9.9 for children attending school aged 5–11 and 12–17, corresponding to a reduction of roughly 35% and 45% compared to the POLYMOD study, but with large differences between countries. Children not attending schools had fewer contacts, typically less than 5 [38]. In Norway, schools were open during the survey but with implementation of IPC measures, which likely reduced contact levels and the risk of transmission. A Norwegian study conducted from the reopening of the schools in August 2020 among children aged 6–16 years suggested limited child-to-child transmission of SARS-CoV-2 in schools [8]. Our reproduction number estimates did not consider depletion of susceptible, age-specific infectivity or susceptibility, or seasonal forcing. It critically assumes that social contact is an adequate surrogate measure of potential transmission events and that the contacts are comparable across the CoMix waves. However, other factors could have affected the risk of transmission during contact, such as personal hygiene measures (e.g., hand washing, face masks), environmental factors (e.g., ventilation, open areas), physical distance with conversational contacts, and the duration of contacts. For example, the potential overestimation of the reproduction number in April, measured against the modelling result, may be related to the early scare and heightened risk perception, which could have affected behaviour during contact. Conversely, the potential underestimation in September may be associated with increasing time spent indoors. However, the information on personal protective behaviour in our study suggested little change between the CoMix waves.

Other findings from the Norwegian CoMix survey have underscored the importance of cognitive and psychological factors in predicting behaviour, in particular identifying predictors of visiting or intending to visit crowded places during the study period [21]. Furthermore, the information gathered through social surveys can be complemented by research utilising non-standard data sources. These may include data streams such as mobile phone tracking to monitor movement patterns [39]. Such multi-faceted research is essential for enhancing our understanding of the dynamic interaction between physical distancing measures, disease prevalence, and population response.

Despite the limitations, our results contribute valuable empirical information into the social behaviour of the Norwegian population during the early COVID-19 pandemic when significant social distancing measures were in place. Social contact data is critical for public health authorities to monitor behaviour, adherence, and have played a pivotal role in developing models used to guide COVID-19 policies. This information will also prove useful in future crisis situations when similar mitigation measures may be required.

### Supplementary Information

Supplementary Material 1.

## Data Availability

The dataset analysed for the study contained anonymized individual-level data. All data were stored securely, and confidentiality was protected in accordance with the Data Protection Act, GDPR and in accordance with requirements of the Norwegian Health Research Act. The anonymised CoMix data used for the analyses will be available on the CoMix platform (https://eur04.safelinks.protection.outlook.com/?url=https%3A%2F%2Fsocialcontactdata.org%2F&data=05%7C02%7CLamprini.Veneti%40fhi.no%7Cdd00e43fb5fd4f1bec7708dc700d2258%7C54475f801baa4ea99185c0de5cc603fe%7C0%7C0%7C638508448919298075%7CUnknown%7CTWFpbGZsb3d8eyJWIjoiMC4wLjAwMDAiLCJQIjoiV2luMzIiLCJBTiI6Ik1haWwiLCJXVCI6Mn0%3D%7C0%7C%7C%7C&sdata=oQYli3TmTQ3oOuw7EhuUVzEOHDaEs12IaBYx3l5CHOQ%3D&reserved=0) soon after the article is published.

## Abbreviations

CI: Confidence intervals
NA: Not available

## References

[1] Nicola M, Alsafi Z, Sohrabi C (2020). The socio-economic implications of the coronavirus pandemic (COVID-19): a review. Int J Surg.

[2] Control ECfDPa. Data on country response measures to COVID-19. 2022. https://www.ecdc.europa.eu/en/publications-data/download-data-response-measures-covid-19. Accessed 5 Sept 2023.

[3] Verelst F, Kuylen E, Beutels P. Indications for healthcare surge capacity in European countries facing an exponential increase in coronavirus disease (COVID-19) cases, March 2020. Euro Surveill. 2020;25(13). 10.2807/1560-7917.ES.2020.25.13.2000323. 10.2807/1560-7917.ES.2020.25.13.2000323 PMC7140594 32265003

[4] Seppälä E, Tønnessen R, Veneti L, Paulsen TH, Steens A, Whittaker R, Bragstad K, Berild JD, Løvlie AL, Naseer U, MacDonald E, Vold L. COVID-19 cases reported to the Norwegian Institute of Public Health in the first six weeks of the epidemic. Tidsskr Nor Laegeforen. 2020;140(18). 10.4045/tidsskr.20.0525. Print 2020 Dec 15. 10.4045/tidsskr.20.0525 33322882

[5] Ursin G, Skjesol I, Tritter J (2020). The COVID-19 pandemic in Norway: the dominance of social implications in framing the policy response. Health Policy Technol.

[6] (DSS) Ds-os. Tidslinje: myndighetenes håndtering av koronasituasjonen. https://www.regjeringen.no/no/tema/Koronasituasjonen/tidslinje-koronaviruset/id2692402/. Accessed 5 Sept 2023.

[7] Johansen TB, Astrup E, Jore S, et al. Infection prevention guidelines and considerations for paediatric risk groups when reopening primary schools during COVID-19 pandemic, Norway, April 2020. Euro Surveill. 2020;25(22). 10.2807/1560-7917.Es.2020.25.22.2000921. 10.2807/1560-7917.ES.2020.25.22.2000921 PMC7336110 32524956

[8] Winje BA, Ofitserova TS, Brynildsrud OB, et al. Comprehensive contact tracing, testing and sequencing show limited transmission of SARS-CoV-2 between children in schools in Norway, August 2020 to May 2021. Microorganisms. 2021;9(12). 10.3390/microorganisms9122587. 10.3390/microorganisms9122587 PMC8705768 34946187

[9] Health NIoP (2020). Seroprevalence of SARS-CoV-2 in the Norwegian population measured in residual sera collected in late summer 2020.

[10] Holmdahl I, Buckee C (2020). Wrong but useful - what covid-19 epidemiologic models can and cannot tell us. N Engl J Med.

[11] Delamater PL, Street EJ, Leslie TF, Yang YT, Jacobsen KH (2019). Complexity of the basic reproduction number (R(0)). Emerg Infect Dis.

[12] Storvik G, Diz-Lois Palomares A, Engebretsen S, Rø G, Engø-Monsen K, Kristoffersen A, de Blasio BF, Frigessi A (2023). A sequential Monte Carlo approach to estimate a time varying reproduction number in infectious disease models: the Covid-19 case. Journal of the Royal Statistical Society Series A: Statistics in Society.

[13] Engebretsen S, Diz-Lois Palomares A, Rø G (2023). A real-time regional model for COVID-19: probabilistic situational awareness and forecasting. PLoS Comput Biol..

[14] Wallinga J, Teunis P, Kretzschmar M (2006). Using data on social contacts to estimate age-specific transmission parameters for respiratory-spread infectious agents. Am J Epidemiol.

[15] Mossong J, Hens N, Jit M (2008). Social contacts and mixing patterns relevant to the spread of infectious diseases. PLoS Med..

[16] Hoang T, Coletti P, Melegaro A (2019). A systematic review of social contact surveys to inform transmission models of close-contact infections. Epidemiology.

[17] Health NIoP. Social contact and vaccine coverage survey, Norway 2016. 2019. https://www.fhi.no/en/cristin-projects/concluded/social-contact-and-vaccine-coverage-survey-norway-2016/. Accessed September 5, 2023.

[18] Verelst F, Hermans L, Vercruysse S (2021). SOCRATES-CoMix: a platform for timely and open-source contact mixing data during and in between COVID-19 surges and interventions in over 20 European countries. BMC Med.

[19] Jarvis CI, Van Zandvoort K, Gimma A (2020). Quantifying the impact of physical distance measures on the transmission of COVID-19 in the UK. BMC Med.

[20] Steens A, Freiesleben de Blasio B, Veneti L, et al. Poor self-reported adherence to COVID-19-related quarantine/isolation requests, Norway, April to July 2020. Euro Surveill. 2020;25(37). 10.2807/1560-7917.ES.2020.25.37.2001607. 10.2807/1560-7917.ES.2020.25.37.2001607 PMC7502884 32945254

[21] Aaro LE, Veneti L, Vedaa O, Smith ORF, De Blasio BF, Robberstad B (2022). Visiting crowded places during the COVID-19 pandemic. A panel study among adult norwegians. Front Public Health.

[22] Statistics Norway. 2020. https://www.ssb.no/en/befolkning/nokkeltall/population. Accessed Jul 2020.

[23] Hale T, Angrist N, Goldszmidt R (2021). A global panel database of pandemic policies (Oxford COVID-19 Government Response Tracker). Nat Hum Behav.

[24] Hens N, Ayele GM, Goeyvaerts N (2009). Estimating the impact of school closure on social mixing behaviour and the transmission of close contact infections in eight European countries. BMC Infect Dis..

[25] Klepac P, Kucharski AJ, Conlan AJ, et al. Contacts in context: large-scale setting-specific social mixing matrices from the BBC pandemic project. medRxiv. 2020:2020.02.16.20023754. 10.1101/2020.02.16.20023754.

[26] Brankston G, Merkley E, Fisman DN (2021). Quantifying contact patterns in response to COVID-19 public health measures in Canada. BMC Public Health.

[27] Coletti P, Wambua J, Gimma A (2020). CoMix: comparing mixing patterns in the Belgian population during and after lockdown. Sci Rep.

[28] Norwegian Institute of Public Health C-mt. Situational awareness and forecasting for Norway, Week 19, May 11 2023. https://www.fhi.no/contentassets/e6b5660fc35740c8bb2a32bfe0cc45d1/vedlegg/nasjonale-og-regionale-rapporter/2023_05_11national_regional_model.pdf. Accessed 5 Sept 2023.

[29] Farrington CP, Whitaker HJ, Wallinga J, Manfredi P (2009). Measures of disassortativeness and their application to directly transmitted infections. Biom J.

[30] Gimma A, Munday JD, Wong KLM (2022). Changes in social contacts in England during the COVID-19 pandemic between March 2020 and March 2021 as measured by the CoMix survey: a repeated cross-sectional study. PLoS Med..

[31] Zhang J, Litvinova M, Liang Y (2020). Changes in contact patterns shape the dynamics of the COVID-19 outbreak in China. Science.

[32] Backer JA, Mollema L, Vos ER, et al. Impact of physical distancing measures against COVID-19 on contacts and mixing patterns: repeated cross-sectional surveys, the Netherlands, 2016-17, April 2020 and June 2020. Euro Surveill. 2021;26(8). 10.2807/1560-7917.Es.2021.26.8.2000994. 10.2807/1560-7917.ES.2021.26.8.2000994 PMC7908067 33632374

[33] Latsuzbaia A, Herold M, Bertemes JP, Mossong J (2020). Evolving social contact patterns during the COVID-19 crisis in Luxembourg. PLoS One..

[34] Feehan DM, Mahmud AS (2021). Quantifying population contact patterns in the United States during the COVID-19 pandemic. Nat Commun.

[35] Dahlen OP, Skirbekk H (2021). How trust was maintained in Scandinavia through the first crisis of modernity. Corp Commun.

[36] Anda EE, Braaten T, Borch KB, et al. Seroprevalence of antibodies against SARS-CoV-2 in the adult population during the pre-vaccination period, Norway, winter 2020/21. Euro Surveill. 2022;27(13). 10.2807/1560-7917.ES.2022.27.13.2100376. 10.2807/1560-7917.ES.2022.27.13.2100376 PMC8973017 35362405

[37] Willem L, Van Hoang T, Funk S, Coletti P, Beutels P, Hens N (2020). SOCRATES: an online tool leveraging a social contact data sharing initiative to assess mitigation strategies for COVID-19. BMC Res Notes.

[38] Wong KLM, Gimma A, Coletti P (2023). Social contact patterns during the COVID-19 pandemic in 21 European countries – evidence from a two-year study. BMC Infect Dis.

[39] Kamineni M, Engo-Monsen K, Midtbo JE, et al. Effects of non-compulsory and mandatory COVID-19 interventions on travel distance and time away from home, Norway, 2021. Euro Surveill. 2023;28(17). 10.2807/1560-7917.ES.2023.28.17.2200382. 10.2807/1560-7917.ES.2023.28.17.2200382 PMC10283475 37103789

