# Supplementary material for: Social contact patterns during the early COVID-19 pandemic in Norway: insights from a panel study, April to September 2020
Source: BMC Public Health. 2024 May 29;24:1438. doi: 10.1186/s12889-024-18853-8 (PMC11137890; doi:10.1186/s12889-024-18853-8)
Supplement: Supplementary file 1 — Supplementary Material 1. [file 12889_2024_18853_MOESM1_ESM.pdf]

Supplementary Material

Article Title:

Social contact patterns during the early COVID-19 pandemic in Norway: insights from a panel study, April to September 2020

Table of Contents

1. Group contacts 2
2. Representativeness of study population 2
3. Control measures overview during the study period 3
4. Social Contact patterns 5

4.1 Additional data on number of contacts 5

4.2 Contact matrices 7

4.3 Sensitivity analysis 13

5. References 16

1. Group contacts

From CoMix wave 2 to CoMix wave 6, 453 participants reported group contacts (449 of those also reported individual contacts); ranging from 11% to 19% of the individuals with contact data per data collection wave. We noticed that some participants reported high numbers with few of them reporting extreme values, such as in CoMix wave 2 where there were two participants that reported 4 847 and 7 021 contacts each. As mentioned in the main article the group contacts were excluded in the main analysis.

As an additional analysis to assess the impact of group contacts in CoMix wave 2 to 6, we calculated the mean number of contacts after fixing the maximum number of group contacts at 50, thereby reducing the influence of participants with exceptionally high contact numbers to the population mean. This affected 12 individuals in wave 2, 16 in wave 3, 8 in wave 4, 16 in wave 5 and 12 in wave 6, all of whom had reported more than 50 group contacts. We present the results from this analysis in Table S1. When comparing these figures with the mean contact numbers reported in Table 2, we observed an increase of 1,2 to 3,1 on average throughout CoMix waves 2 to 6. Similar as in Table 2, the lowest number of contacts was reported in wave 4 and the highest in wave 3.

Table S1: Summary of daily individual and group contacts reported in each CoMix wave in 2020. The crude mean of contacts (not weighted) per wave is presented in the last column.

CoMix Wave	Participants	Group contacts	Individual contacts	Total contacts	Crude mean of contacts
**Wave 1**	1 400	N.A.*	5 360	5 360	3.8
**Wave 2**	1 182	2 586	4 477	7 063	6.0
**Wave 3**	1 012	3 020	4 041	7 061	7.0
**Wave 4**	931	1 144	3 011	4 155	4.5
**Wave 5**	768	1 839	2 850	4 689	6.1
**Wave 6**	645	1 678	2 335	4 013	6.2

**N.A.: Not available. In wave 1, participants were not able to report group contacts.*

***Total contacts= Sum of group and individual contacts.*

2. Representativeness of study population

Our participants were sampled to be representative by age, gender, and region of residence. The dropouts and new recruitment of participants impacted a little in the composition of the sample with only small changes in the representativeness (Table S2).

We observed that the representativeness by gender and county was quite good. Regarding the age distribution, the 18-29 age group were undersampled ranging from 7 to 16% across the CoMix waves compared to the 20% representation in the general population. Conversely, the 50-69 age group showed a slight oversampling, ranging from 35 to 40% through the waves in contrast to the 30% representation in the general population. In addition, participants residing in Oslo County were marginally overrepresented throughout the waves, constituting 17-18% of the sample compared to the 13% presentation in the general population. While these variances were considered minor for the purpose of our study, we decided to conduct weighted analysis for the contact pattern data to account for these differences.

Table S2: Sample characteristics in the baseline survey and of each CoMix wave (starting date of each CoMix data collection wave is indicated in brackets) compared to the proportions of the 2020 Norwegian adult population.

**Demographic characteristics**	**Number of participants per dataset (%)**							**% of the adult Norwegian population 2020**
	**Baseline survey, 2017 n=309**	**Wave 1 (24 April), n=1400**	**Wave 2 (19 May), n=1182**	**Wave 3 (9 June), n=1012**	**Wave 4 (21 July), n=931**	**Wave 5 (25 Aug.), n=768**	**Wave 6 (23 Sept.), n=645**	
**Gender**								
Male	146 (47 %)	702 (50 %)	610 (52 %)	534 (53 %)	487 (52 %)	421 (55 %)	357 (55 %)	50 %
Female	163 (53 %)	698 (50 %)	569 (48 %)	475 (47 %)	441 (47 %)	345 (45 %)	287 (45 %)	50 %
NA	0	0	3(-)	3(-)	3(-)	2 (-)	12 (-)	-
**Age group**								
18-29	46 (15 %)	217 (16 %)	121 (10 %)	98 (10 %)	78 (8 %)	57 (7 %)	70 (11 %)	20 %
30-49	36 (12 %)	509 (36 %)	410 (35%)	352 (35 %)	300 (32 %)	257 (34 %)	210 (33 %)	34 %
50-69	93 (30 %)	493 (35 %)	458 (39%)	406 (40 %)	406 (44 %)	319 (42 %)	262 (41 %)	30 %
70+	134 (43 %)	181 (13 %)	193 (16 %)	156 (15 %)	147 (16 %)	135 (18 %)	103 (16 %)	16 %
**County**								
Agder	NA	70 (5 %)	57 (5 %)	53 (5 %)	42 (5 %)	41 (5 %)	29 (5 %)	6 %
Innlandet	NA	61 (4 %)	51 (4 %)	46 (5 %)	54 (6 %)	35 (5 %)	28 (4 %)	7 %
Møre og Romsdal	NA	66 (5 %)	58 (5 %)	47 (5 %)	49 (5 %)	42 (6 %)	38 (6 %)	5 %
Nordland	NA	50 (4 %)	47 (4 %)	43 (4 %)	32 (3 %)	24 (3 %)	24 (4 %)	5 %
Oslo	NA	254 (18 %)	209 (18 %)	186 (18 %)	156 (17 %)	132 (17 %)	113 (18 %)	13 %
Rogaland	NA	124 (9 %)	105 (9 %)	88 (9 %)	82 (9 %)	75 (10 %)	66 (10 %)	9 %
Troms og Finnmark	NA	62 (4 %)	44 (4 %)	42 (4 %)	36 (4 %)	37 (5 %)	21 (3 %)	5 %
Trøndelag	NA	131 (9 %)	110 (9 %)	90 (9 %)	73 (8 %)	58 (8 %)	52 (8 %)	9 %
Vestfold og Telemark	NA	114 (8 %)	94 (8 %)	79 (8 %)	81 (9 %)	61 (8 %)	53 (8 %)	8 %
Vestland	NA	167 (12 %)	136 (12 %)	113 (11 %)	111 (12 %)	82 (11 %)	80 (12 %)	12 %
Viken	NA	301 (22 %)	271 (23 %)	225 (22 %)	215 (23 %)	181 (24 %)	141 (22 %)	23

3. Control measures overview during the study period

In Norway, the stringency of control measures implemented since the beginning of the pandemic varied depending on the evolving epidemiological situation. In Table 3, we present some of the social distance measures implemented by the Norwegian government during the data collection period of the CoMix study (1, 2). We should note that the social distance measures included in Table S3 are referring to the national guidelines, and there may have been variations in local measures may varied in some instances.

In Norway, children's daycare and schools were closed on 13 March 2020, and reopened under strict IPC measures from 20 April 2020. From end of May, a traffic light model was developed to guide administrators on IPC strategies in primary schools (grade 1–7, children 6–13 years of age) (3, 4). This three-tiered system, with non-pharmaceutical measures, depended on local incidence and infection pressure. The guidelines advised the establishment of cohorts consisting of small permanent groups of children and staff with limited interaction between cohorts, alongside timely testing and isolation of symptomatic cases, and tracing and quarantine of their contacts. During the CoMix study period, the schools for children below 18 years old were all closed in wave 1 (lockdown) and 4 (summer holidays). Primary schools had strict IPC measures in wave 2 and the 'traffic model' restrictions in wave 3, 5 and 6. All other schools had some IPC measures in place even though they were open for the rest of the waves. Moreover, individual companies may have implemented their own distinct measures.

Table S3: Control measures implemented during the week of the CoMix data collection (data collection dates are indicated in brackets) and COVID-19 weekly incidence in Norway, April-September 2020.

	**Data collection period**						**Dates during CoMix Study that the measures were implemented**
**Control measures**	**Wave 1, 24-30 April**	**Wave 2, 19-26 May**	**Wave 3, 9-16 June**	**Wave 4, 21-27 July**	**Wave 5, 25 Aug. -2 Sept.**	**Wave 6, 23-30 Sept.**	
Ban of all events	Yes	No	No	No	No	No	12/3/2020-6/5/2020
Closure of kindergartens/daycare	No	No	No	Summer break	No	No	12/3/2020-20/4/2020
Closure of primary schools-	Yes until 26 April	No	No, but with restrictions*	Summer break	No, but with restrictions*	No, but with restrictions*	12/3/2020-26/4/2020 Open but with traffic light model restrictions after 29/05/2020*
Closure of secondary schools	Yes	No	No	Summer break	No	No	12/3/2020-11/5/2020
Closure of higher education/universities	Yes	Yes	Yes until 14th June	Summer break	No	No	12/3/2020-14/6/2020
Closure of cafes-restaurants	Yes	Yes	No, but with restrictions**	No, but with restrictions**	No, but with restrictions**	No, but with restrictions**	12/3/2020-31/5/2020, Open but with restrictions** after 1/6/2020
Closure of pubs/bars	Yes	Yes	No, but restrictions applied**	No, but with restrictions**	No, but with restrictions**	No, but with restrictions**	12/3/2020-31/5/2020, Open but with restrictions** after 1/6/2020
Closure of gyms, sports centres	Yes	No	No	No	No	No	12/3/2020-14/5/2020
Any gatherings above 50, indoors or outdoors not allowed	Yes	No	No	No	No	No	7/5/2020-14/6/2020
Teleworking strongly suggested	Yes	Yes	Yes	No	No	No	10/3/2020-17/6/2021
Private gathering restrictions	Yes (Advice to not meet in groups >5)	Yes (Advice to not meet in groups >20)	Yes (Advice to not meet in groups >20)	Yes (Advice to not meet in groups >20)	Yes (Advice to not meet in groups >20)	Yes (Advice to not meet in groups >20)	From 12/3 advised not to meet in groups of more than 5. Also advised to meet outdoors. From 7/5 advice to avoid gathering >20 people in private homes.
**Weekly Reported cases among all Norwegian population (sampling week)**	359 (week 17)	101 (week 21)	80 (week 24)	94 (week 30)	374 (week 35)	776 (week 39)	

**Note: From end of May, a traffic light model was developed to guide administrators on IPC strategies in primary schools (grade 1–7, children 6–13 years of age.*

***Note: From 1st of June, cafes/restaurants/bars could reopen as long as they could provide a distance of one meter between guests and seating for all guests. All places had to close by 24.00..*

4. Social Contact patterns

4.1 Additional data on number of contacts

Fig. S1: Mean number of daily contacts reported,with 95% confidence intervals, that occurred A) outdoors and B) indoors, stratified by participant age group and data collection period (CoMix wave). The starting date of each CoMix data collection wave is indicated in the brackets. These results are weighted by gender.

A)

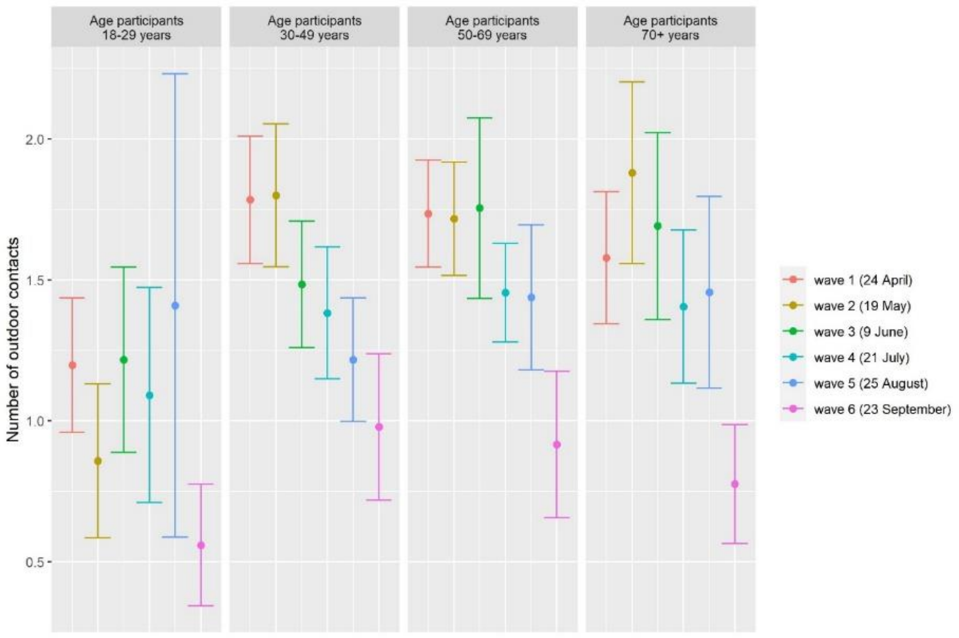

B)

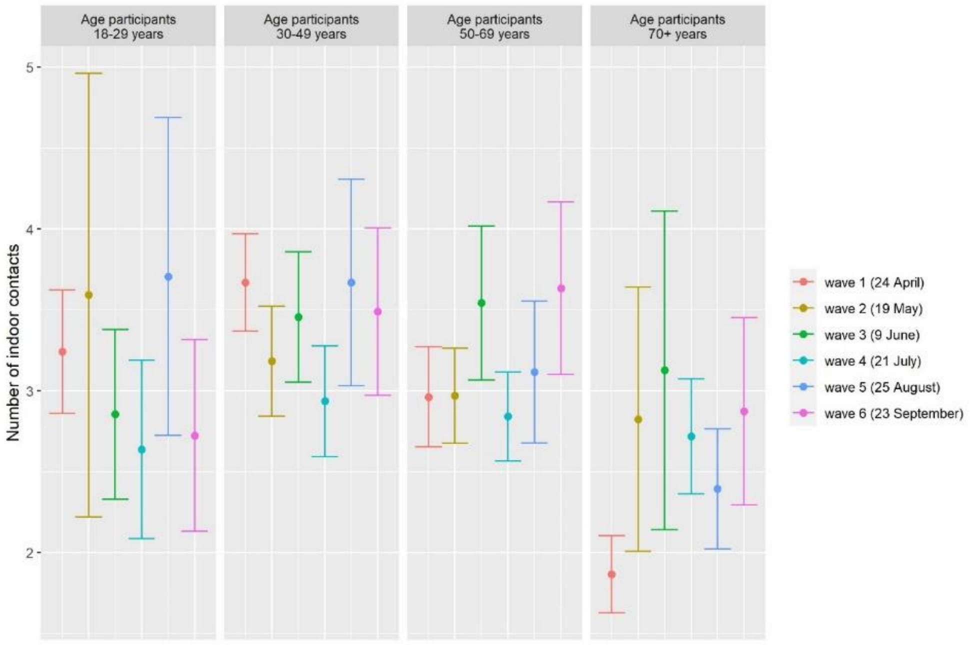

Table S4: Percentage (crude) of contacts that all participants reported by place* and type of contact in each CoMix wave. The starting date of each CoMix data collection wave is indicated in the brackets.

Place of contact and type of contact		*Wave 1, (24 April), n=1400*		*Wave 2, (19 May), n=1182*		*Wave 3, (9 June), n=1012*		**Wave 4, (21 July), n=931**		**Wave 5, (25 Aug.),` n=768**		**Wave 6, (23 Sept.,) n=645**	
		Total contacts	%	Total contacts	%	Total contacts	%	Total contacts	%	Total contacts	%	Total contacts	%
Place of contact	Home	2 457	**41%**	1 906	**37%**	1 401	**31%**	1 369	**38%**	1 075	**33%**	821	**31%**
	Other house	471	**8%**	566	**11%**	318	**7%**	397	**11%**	256	**8%**	230	**9%**
	Work	1 206	**20%**	784	**15%**	1 284	**28%**	390	**11%**	669	**21%**	714	**27%**
	Supermarket-shops	508	**8%**	682	**13%**	525	**12%**	499	**14%**	360	**11%**	262	**10%**
	Outside other (parks-countryside)	642	**11%**	490	**10%**	339	**7%**	270	**8%**	232	**7%**	115	**4%**
	Public_transport	104	**2%**	137	**3%**	116	**3%**	143	**4%**	86	**3%**	77	**3%**
	School	90	**1%**	104	**2%**	52	**1%**	18	**1%**	120	**4%**	64	**2%**
	Leisure (bars, restaraunts etc)	31	**1%**	131	**3%**	118	**3%**	157	**4%**	85	**3%**	85	**3%**
	Sport	66	**1%**	32	**1%**	42	**1%**	33	**1%**	107	**3%**	66	**3%**
	Other	466	**8%**	323	**6%**	357	**8%**	318	**9%**	266	**8%**	204	**8%**
	**Total, all places[a]**	**6 041**	**100%**	**5 155**	**100%**	**4 552**	**100%**	**3 594**	**100%**	**3 256**	**100%**	**2 638**	**100%**
Physical contact		1 707	**32%**	1 307	**29%**	1 055	**26%**	1 062	**35%**	811	**28%**	672	**29%**
Contact indoors[b]		4 389	**65%**	3 630	**64%**	3 407	**68%**	2 635	**67%**	2 452	**70%**	2 172	**79%**
Contact outdoors		2 325	**34%**	2 007	**36%**	1 611	**32%**	1 296	**33%**	1 042	**30%**	576	**21%**

a: A contact could have been made in more than one place at the same day. Therefore, the proportions were calculated using the total of contacts for all places reported.

b: A contact could have been made in more than one place at the same day (outdoors and indoors). Therefore, the proportions were calculated using the total of contact for both places.

Fig. S2: Percentage (crude) of daily contacts reported by location* of participants in each CoMix wave. The starting date of each CoMix data collection wave is indicated in the brackets.

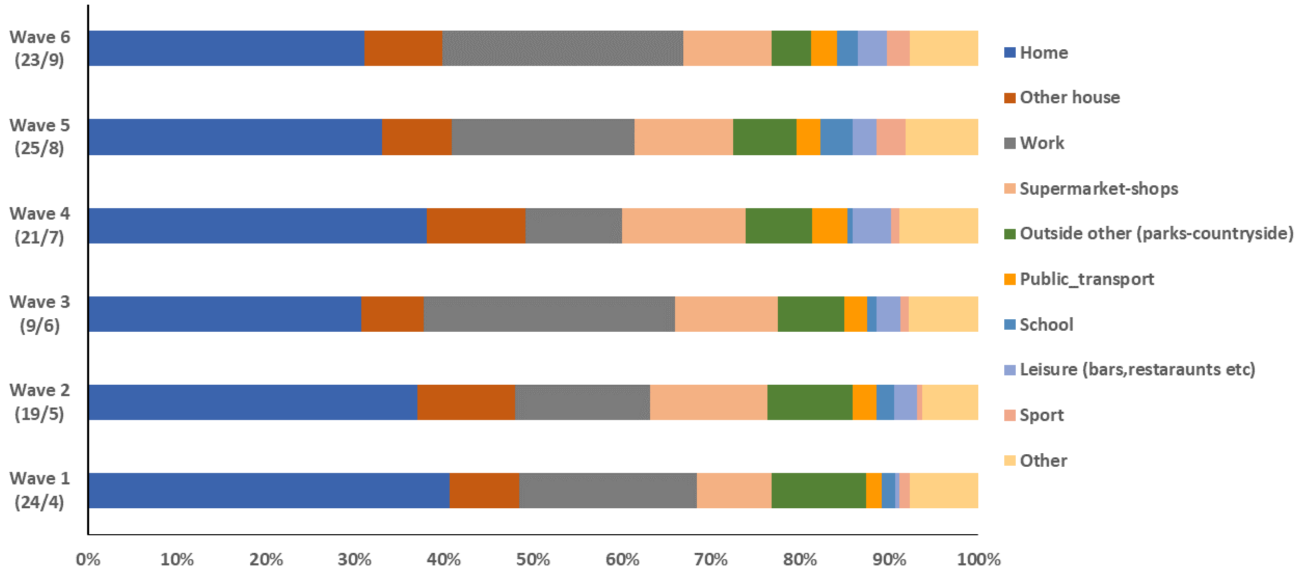

**Note: A contact could have been made in more than one place at the same day. Therefore, the proportions here were calculated using the total of contacts for all places reported.*

4.2 Contact matrices

Fig S3: Boxplots of the estimated ratio of the maximum eigenvalues (CoMix/Baseline) of the adult-to-adult contract matrices for each CoMix wave by location and type of contact. The ratios of dominant eigenvalues represent bootstrap sample pairs (N=10 000) that were used in the imputation process to scale contacts of children in the baseline contact matrices.

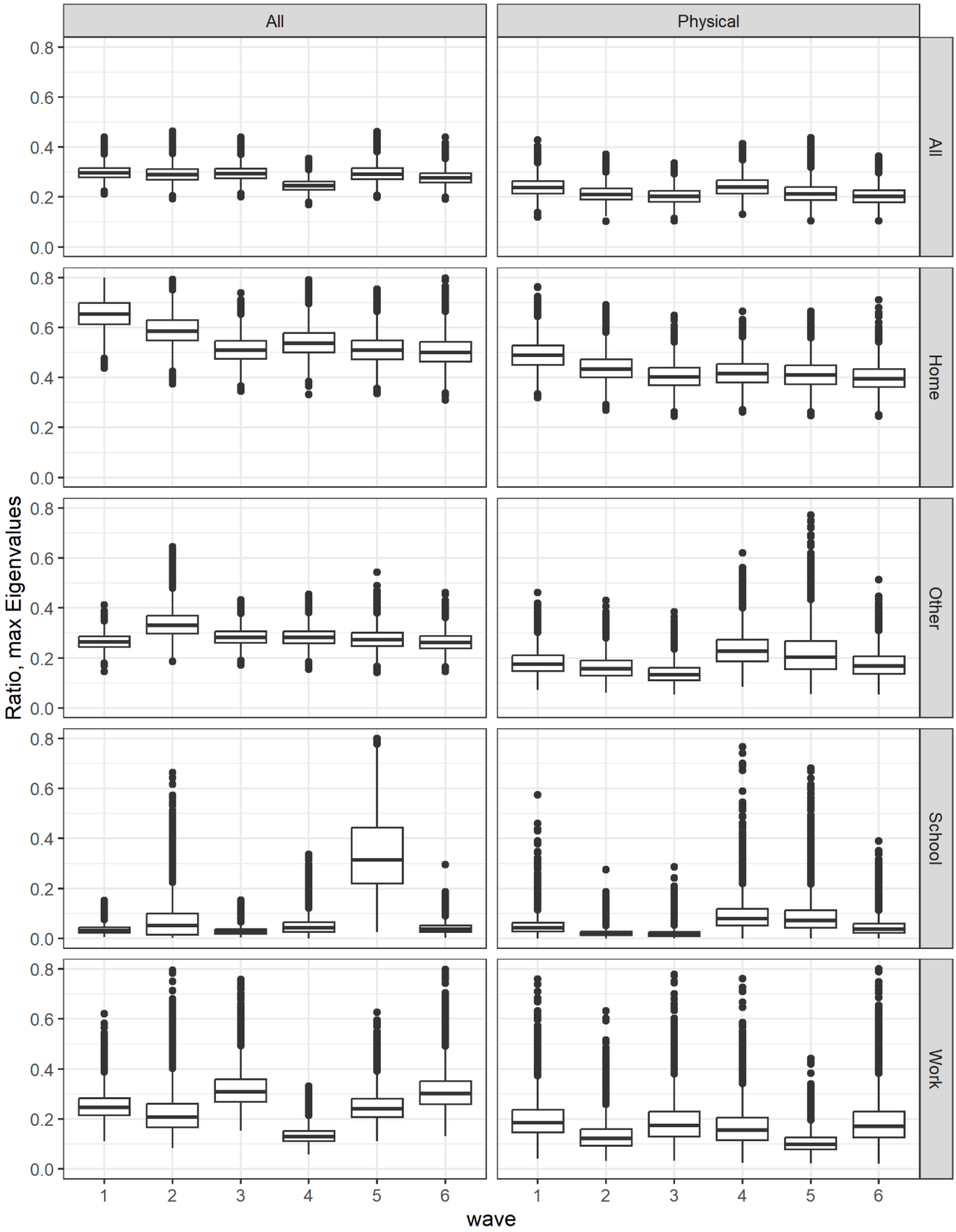

Fig S4: Boxplots of the estimated ratio of the maximum eigenvalues (CoMix/Baseline) of the full population matrices for each CoMix wave by location and type of contact. The ratios of dominant eigenvalues represent bootstrap sample pairs (N=10 000).

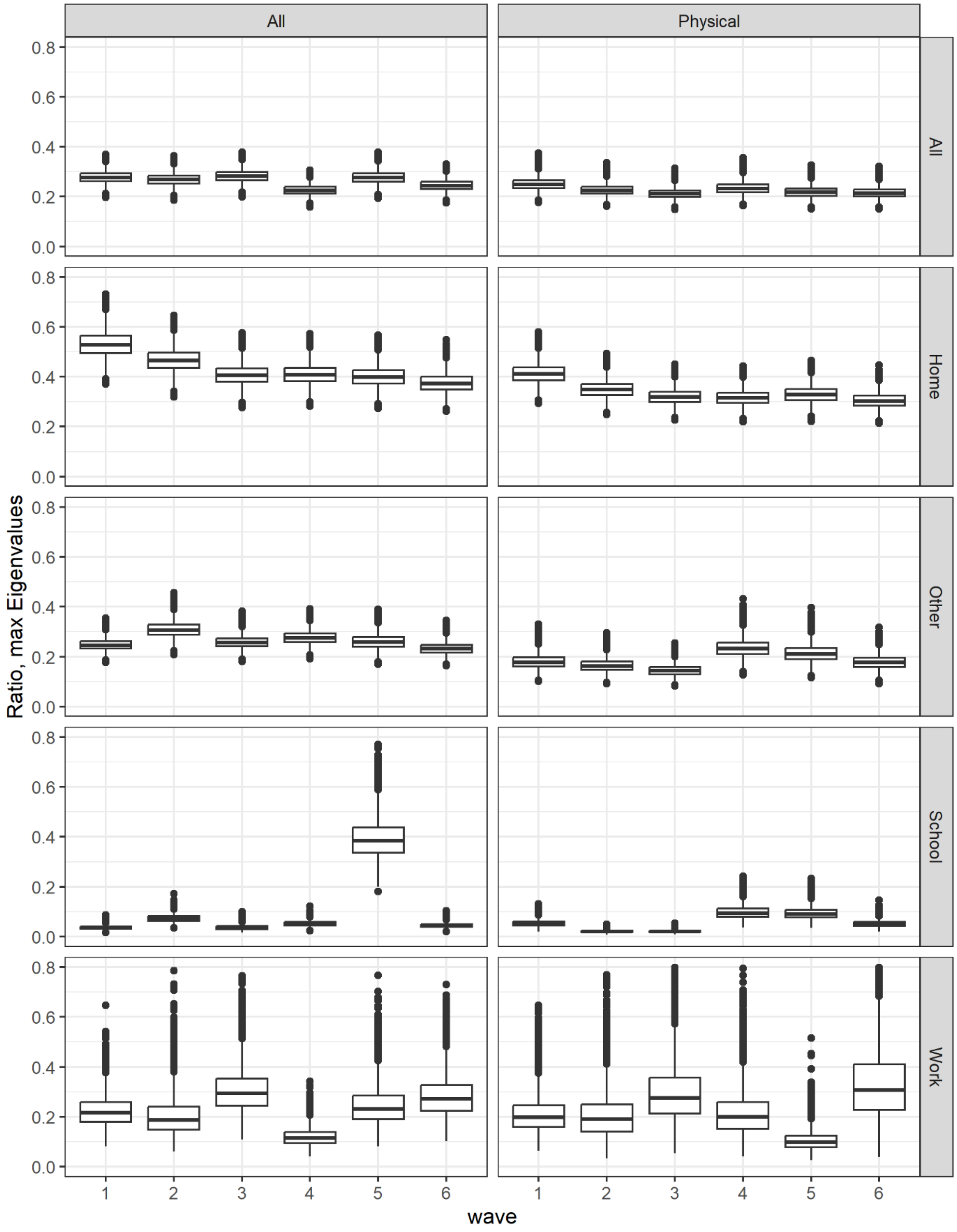

Fig S5: Imputed social contact matrices showing the mean number of daily physical contacts in the six CoMix waves; the corresponding matrix from the 2017 baseline survey is shown below as a reference. The matrices report bootstrap mean values from N=10 000 samples. Data were weighted on age and adjusted for reciprocity of contacts.

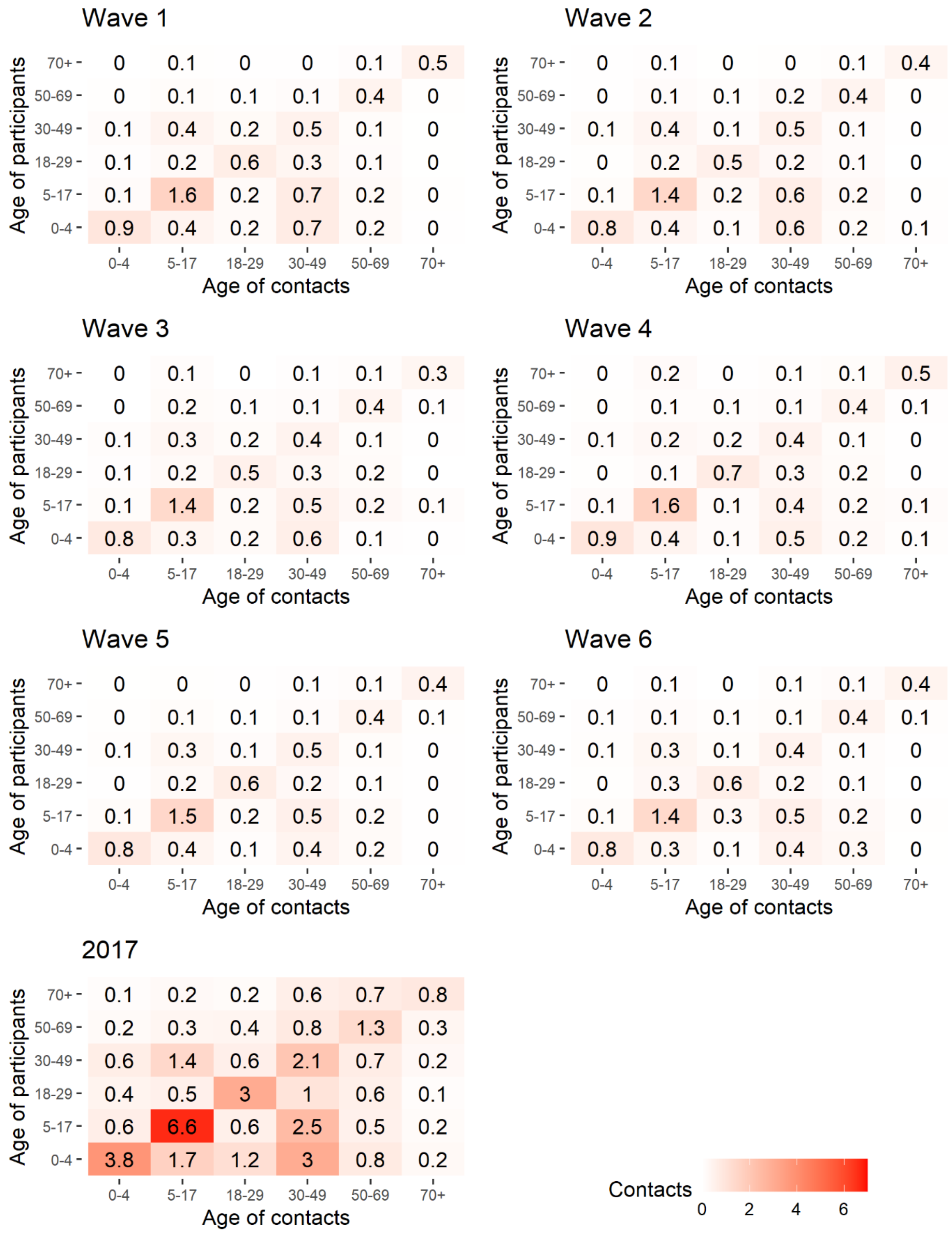

Fig S6: Social contact matrices of the six CoMix waves showing the mean number of daily contacts reported; below is shown the corresponding matrix of contacts reported by adults in the 2017 baseline survey as a reference. The figures represent bootstrap mean values from N=10 000 samples. Data were weighted on age. Note: Data on children contacts were not available (noted as NA: not available) for the CoMix waves and were available for the 2017 survey but were not included (noted as NS: not shown) in these matrices).

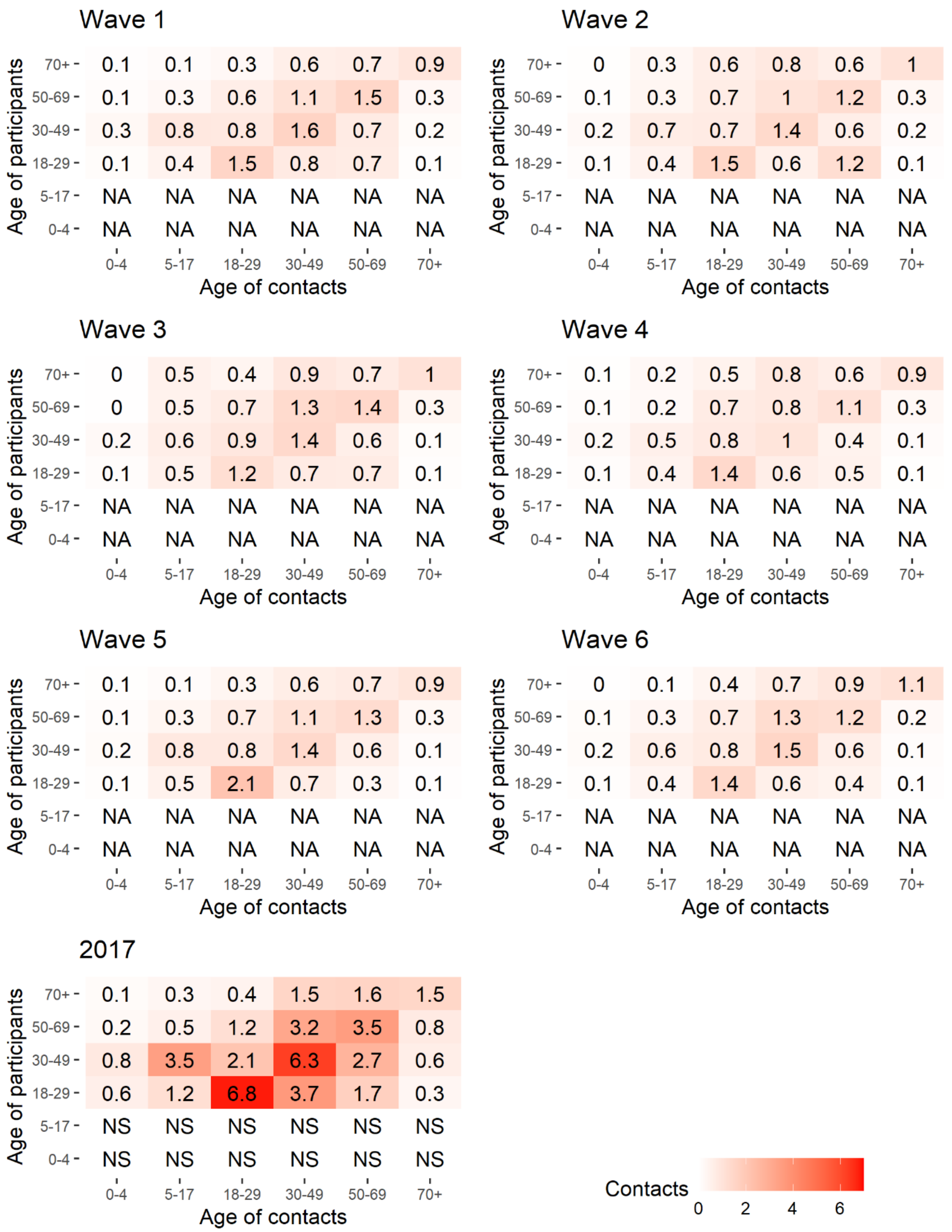

Fig S7: Social contact matrices of the six CoMix waves showing the mean number of daily physical contacts reported; below is shown the corresponding matrix of physical contacts reported by adults in the 2017 baseline survey as a reference. The matrices represent bootstrap mean values from N=10 000 samples. Data were weighted on age. Note: Data on children contacts were not available (noted as NA: not available) for the CoMix waves and were available for the 2017 survey but were not included (noted as NS: not shown) in these matrices).

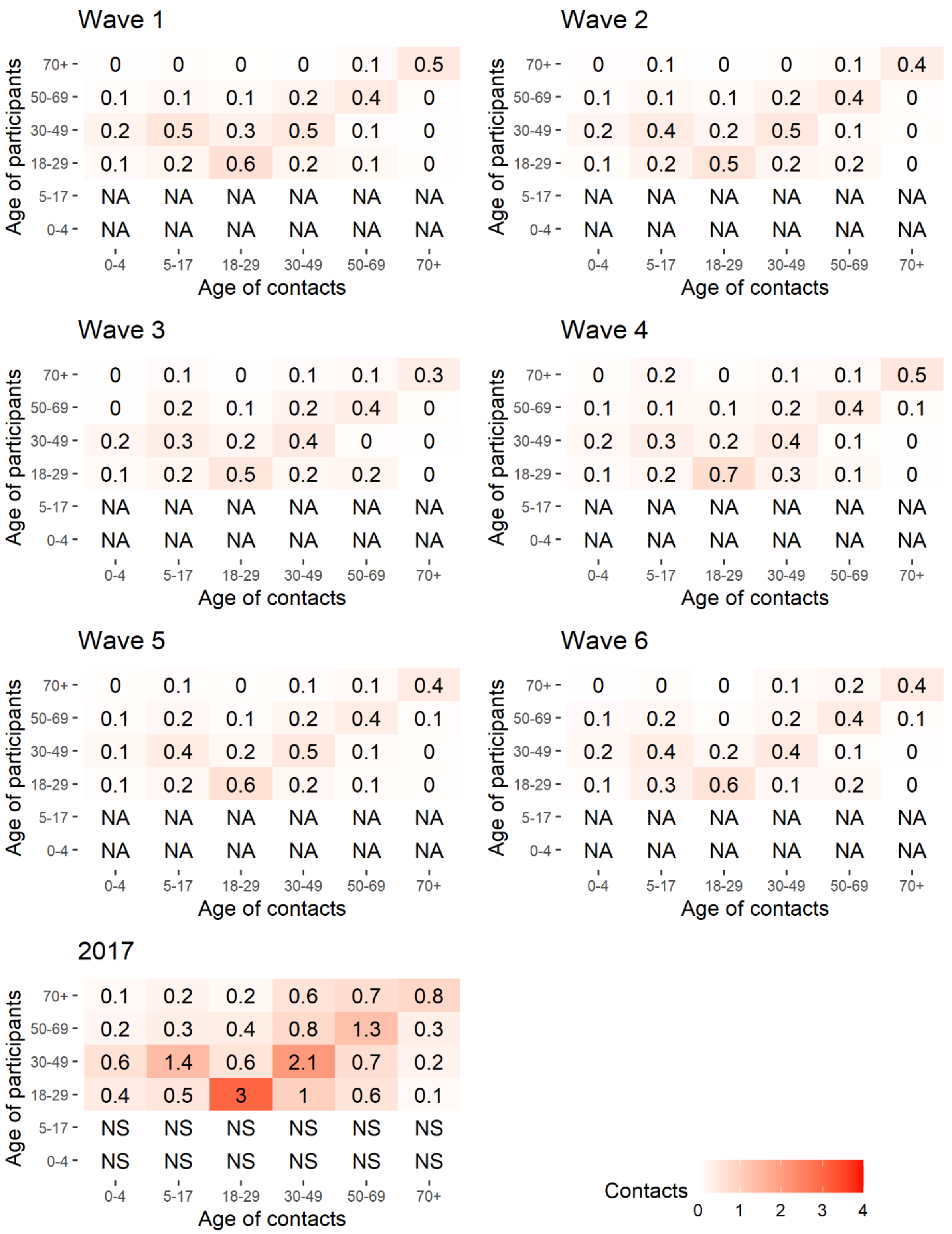

Fig S8: Imputed, setting-specific social contact matrices showing the mean number of daily physical contact for the six CoMix waves. Locations include all contacts made in the home, at schools, at workplaces and other community contacts (transport, sport activities etc.) The figures represent bootstrap mean values of N=10 000. Data were weighted on age and adjusted for reciprocity of contacts.

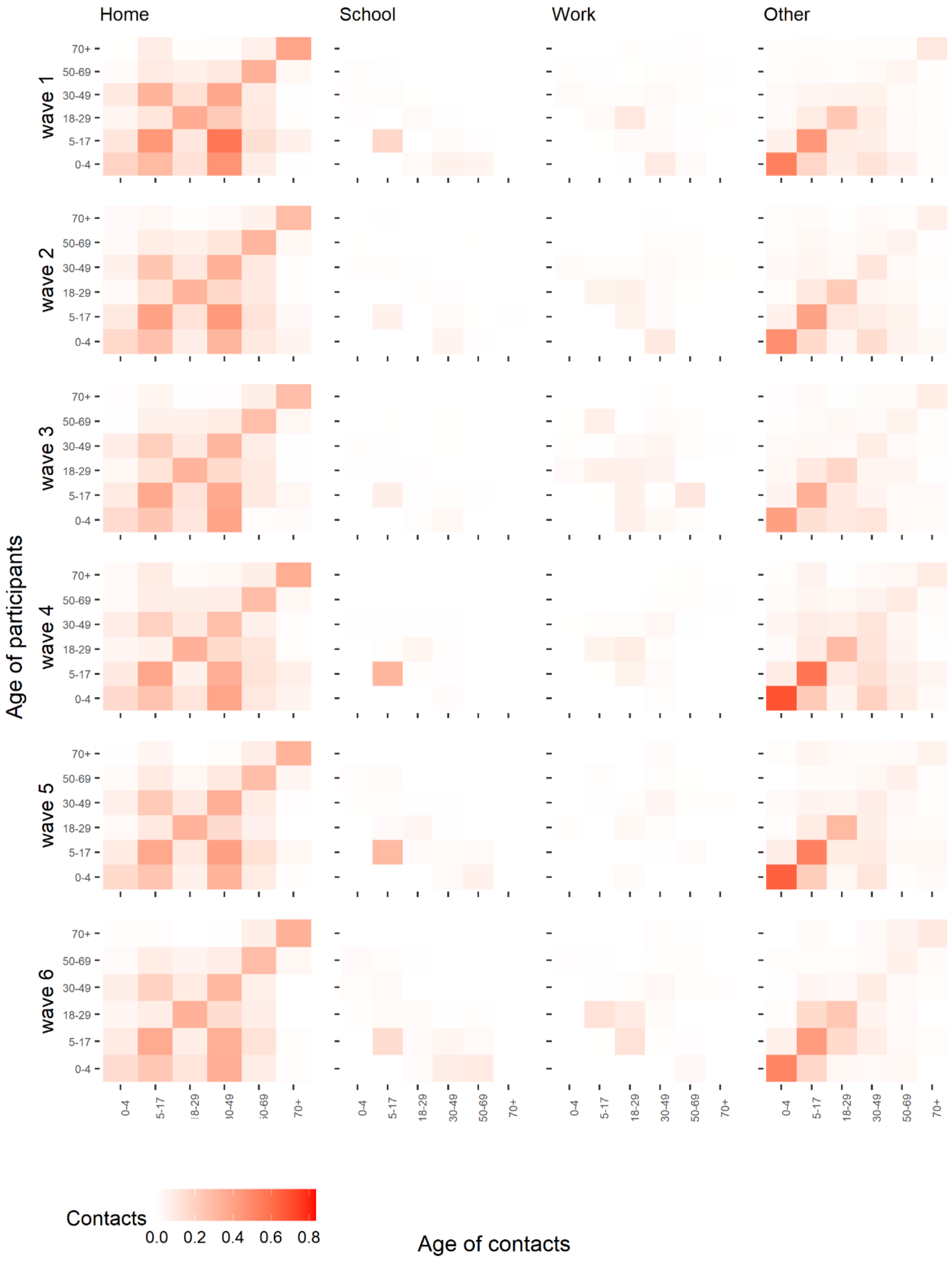

Table S5: Disassortativity measures from 2017 baseline survey and CoMix waves by type and location: standardized indices (I_S^2) with interquartile bootstrap intervals (IQR) of imputed matrices.

	Location	2017 Baseline (I_S^2) mean (Q1-Q3)	Wave 1 (I_S^2) mean (Q1-Q3)	Wave 2 (I_S^2) mean (Q1-Q3)	Wave 3 (I_S^2) mean (Q1-Q3)	Wave 4 (I_S^2) mean (Q1-Q3)	Wave 5 (I_S^2) mean (Q1-Q3)	Wave 6 (I_S^2) mean (Q1-Q3)
All	All	0.43 (0.42-0.44)	0.46 (0.46-0.47)	0.55 (0.53-0.56)	0.59 (0.57-0.61)	0.53 (0.52-0.54)	0.46 (0.44-0.47)	0.48 (0.46-0.49)
	Home	0.63 (0.61-0.65)	0.52 (0.51-0.53)	0.51 (0.50-0.52)	0.48 (0.46-0.49)	0.6 (0.58-0.61)	0.49 (0.48-0.51)	0.47 (0.45-0.52)
	School	0.19 (0.18-0.20)	0.47 (0.38-0.56)	0.63 (0.27-0.88)	0.31 (0.27-0.35)	0.08 (0.06-0.09)	0.17 (0.09-0.22)	0.55 (0.31-0.74)
	Work	0.36 (0.33-0.39)	0.34 (0.32-0.35)	0.52 (0.43-0.60)	0.68 (0.54-0.80)	0.32 (0.29-0.34)	0.40 (0.35-0.42)	0.38 (0.34-0.42)
	Other	0.46 (0.45-0.48)	0.47 (0.45-0.48)	0.57 (0.53-0.60)	0.51 (0.49-0.53)	0.53 (0.50-0.55)	0.47 (0.44-0.49)	0.44 (0.42-0.44)
Physical	All	0.50 (0.48-0.51)	0.47 (0.46-0.48)	0.50 (0.48-0.51)	0.54 (0.51-0.56)	0.55 (0.53-0.57)	0.46 (0.44-0.48)	0.51 (0.48-0.53)
	Home	0.63 (0.6-0.65)	0.57 (0.55-0.58)	0.59 (0.57-0.60)	0.57 (0.55-0.58)	0.65 (0.63-0.67)	0.56 (0.55-0.58)	0.58 (0.56-0.60)
	School	0.19 (0.17-0.21)	0.67 (0.53-0.80)	0.77 (0.67-0.87)	0.44 (0.36-0.52)	0.09 (0.06-0.11)	0.55 (0.26-0.76)	0.88 (0.50-1.17)
	Work	0.33 (0.29-0.36)	0.60 (0.52-0.67)	0.38 (0.24-0.49)	0.77 (0.43-1.03)	0.22 (0.19-0.25)	0.52 (0.34-0.66)	0.35 (0.25-0.43)
	Other	0.52 (0.5-0.55)	0.43 (0.39-0.46)	0.5 (0.47-0.57)	0.46 (0.42-0.50)	0.54 (0.50-0.58)	0.45 (0.40-0.50)	0.34 (0.30-0.38)

4.3 Sensitivity analysis

In this sensitivity analysis, we estimate the impact of varying the children-to-children contacts on the estimated R0-values. We reduced the contacts for the (0-4, 5-17) age groups by 25%, 50%, 65% and 80% relative to the contacts in the 2017 baseline study. All other contacts remained unchanged. We re-estimated the R0 ratios for each survey wave and for all contacts and physical contacts.

Fig S9: Comparison of CoMix and baseline dominant eigenvectors and eigenvalues in four different assumption scenarios regarding the magnitude of reduction among children contacts; children-to-children contacts reduced by 25%, 50%, 65% and 80%: (A) Dominant normalised eigenvectors from contact matrices of all contacts by age group, (B) Dominant normalised eigenvectors from contact matrices of physical contacts by age group.

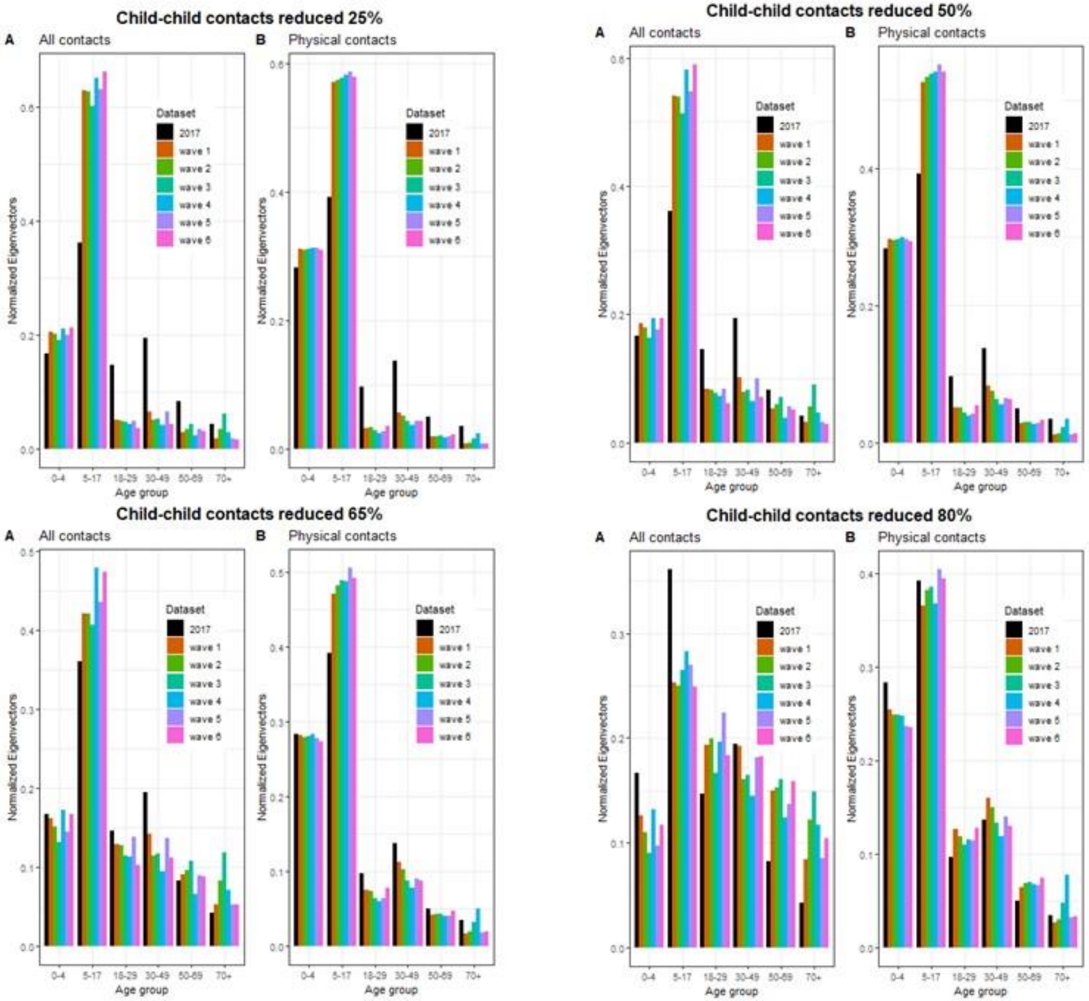

Fig S10: Boxplots showing the estimated basic reproduction numbers, R0, derived from the six CoMix wave surveys in four different assumption scenarios regarding the magnitude of reduction among children contacts; children-to-children contacts reduced by 25%, 50%, 65% and 80%. The R0 values were calculated based on contact matrices that consider all contacts (left panel) and exclusively physical contacts (right panel). The estimation involves multiplying the maximum eigenvalue ratios (Imp CoMix/baseline) by an initial R0-value established for Norway before the March 2020 lockdown.

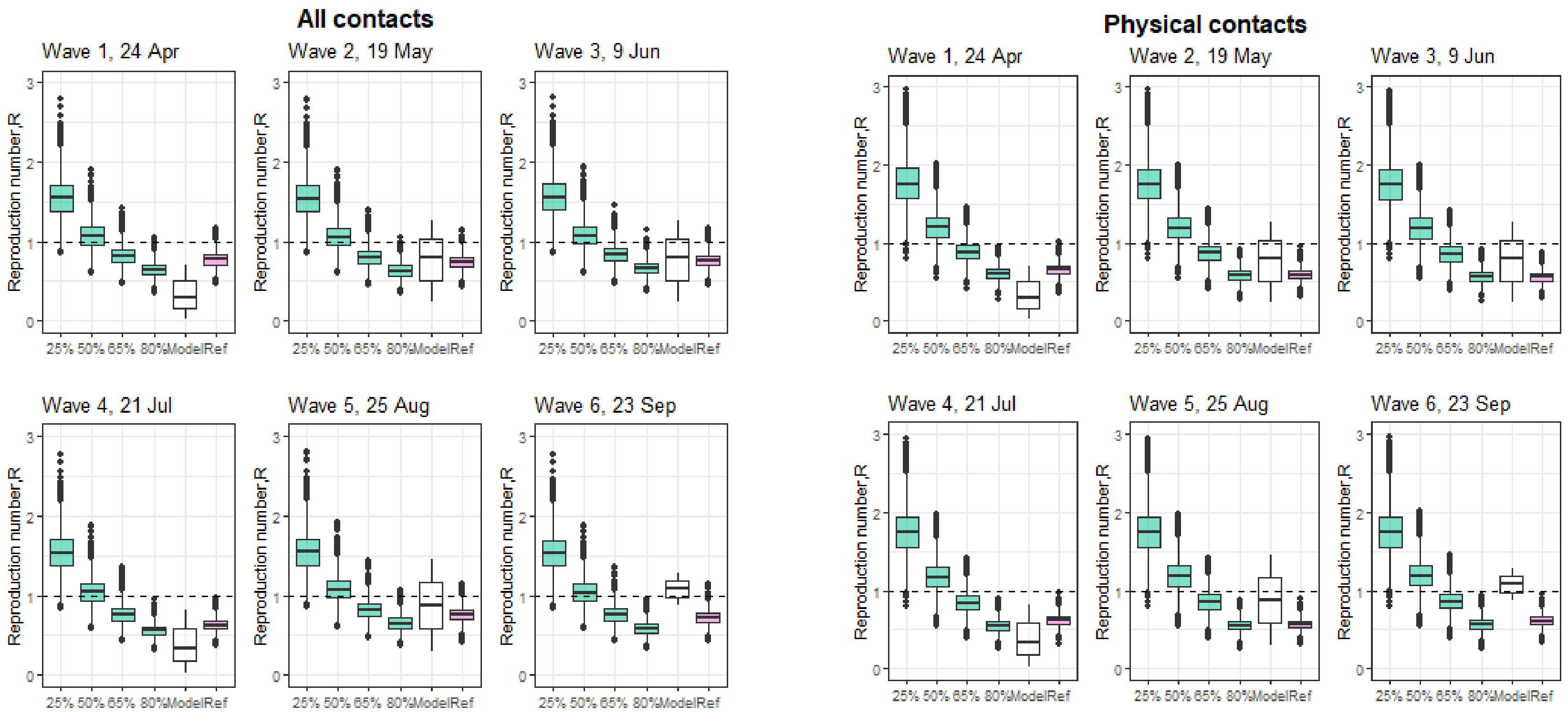

5. References

1. Ursin G, Skjesol I, Tritter J. The COVID-19 pandemic in Norway: The dominance of social implications in framing the policy response. Health Policy Technol. 2020;9(4):663-72. doi:10.1016/j.hlpt.2020.08.004
2. (DSS) Ds-os. Tidslinje: myndighetenes håndtering av koronasituasjonen. https://www.regjeringen.no/no/tema/Koronasituasjonen/tidslinje-koronaviruset/id2692402/
3. Johansen TB, Astrup E, Jore S, et al. Infection prevention guidelines and considerations for paediatric risk groups when reopening primary schools during COVID-19 pandemic, Norway, April 2020. Euro Surveill. 2020;25(22). doi:10.2807/1560-7917.Es.2020.25.22.2000921
4. Winje BA, Ofitserova TS, Brynildsrud OB, et al. Comprehensive Contact Tracing, Testing and Sequencing Show Limited Transmission of SARS-CoV-2 between Children in Schools in Norway, August 2020 to May 2021. Microorganisms. 2021;9(12). doi:10.3390/microorganisms9122587
